# Bioinspired Auxetic Metastructures Enable Biomechanically Adaptive, Machine Learning-Enhanced Self-Powered Sensing with Ultrahigh Efficiency

**DOI:** 10.1007/s40820-026-02125-8

**Published:** 2026-03-18

**Authors:** Wei Wang, Xuechuan Wang, Linbin Li, Yi Zhou, Wenlong Zhang, Long Xing, Long Xie, Yitong Wang, Ouyang Yue, Xinhua Liu

**Affiliations:** 1 College of Bioresources Chemical and Materials Engineering, Shaanxi University of Science & Technology, Xi’an, 710021 People’s Republic of China; 2 College of Chemistry and Chemical Engineering, Shaanxi University of Science & Technology, Xi’an, 710021 People’s Republic of China

**Keywords:** Tissue–device matching, Adaptive self-powered sensors, Auxetic effect, Energy conversion efficiency, Neural network model

## Abstract

Self-powered flexible sensors exhibit revolutionary potential in next-generation wearable technologies owing to their exceptional sensitivity and self-sustaining energy harvesting capabilities. Nevertheless, their widespread deployment remains constrained by three fundamental challenges: dynamic mechanical mismatch between biological tissues and rigid devices, suboptimal energy conversion efficiency, and interfacial impedance fluctuation under deformation. Drawing inspiration from the unique negative Poisson’s ratio mesh architecture of lacewing wings, we present a bioinspired auxetic metastructure-engineered triboelectric nanogenerator. This innovative design integrates engineered collagen and micropatterned fluorinated ethylene propylene as triboelectric layers, unified by an auxetic framework with re-entrant hexagonal unit cells interconnected via triangular ligaments. The metastructure enables exceptional lateral expansion under longitudinal strain while simultaneously enhancing structural rigidity and deformation adaptability. This dual functionality effectively minimizes tissue–device mechanical mismatch, thereby significantly improving signal fidelity, sensitivity, and mechanical-to-electrical conversion efficiency during multi-axial deformations. The optimized device achieves remarkable performance metrics, delivering 478 V output voltage with 13.8% energy conversion efficiency in linear configuration, while demonstrating threefold enhanced stability (58 V, 7.58% efficiency) under complex bending compared to conventional designs. Integrated with a convolutional neural network-based machine learning enables exceptional classification accuracy (> 99%) across diverse material recognition tasks, validating its robustness as a next-generation platform for adaptive self-powered wearable sensing.

**Supplementary Information:**

The online version contains supplementary material available at 10.1007/s40820-026-02125-8.

## Highlights

- Bioinspired auxetic triboelectric nanogenerator utilizes negative Poisson’s ratio to resolve interfacial mechanical mismatch. A “conformal self-adaptation” mechanism via synclastic curvature maximizes contact area and signal stability on curvilinear surfaces.
- The optimized structure achieves a 3.2-fold increase in bending-mode energy conversion efficiency compared to non-auxetic controls, ensuring robust energy harvesting performance under dynamic deformation.
- An integrated self-powered sensor array coupled with a convolutional neural network deep learning model enables intelligent object recognition with 98.7% accuracy, demonstrating precise human–machine interaction capabilities.

**Supplementary Information:**

The online version contains supplementary material available at 10.1007/s40820-026-02125-8.

## Graphical abstract

**Figure:**
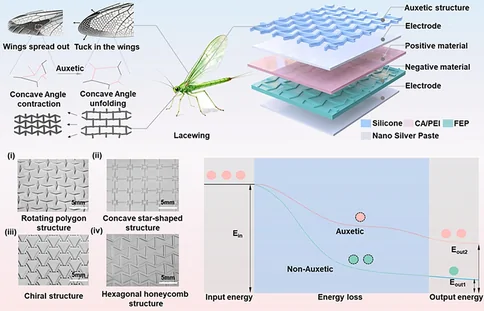

## Introduction

Flexible sensors, as key components of the Internet of Things (IoT) and personalized healthcare systems, hold tremendous potential for real-time health monitoring, human–machine interfaces, and motion posture analysis [1–4]. Unlike conventional passive sensors that merely detect signals and rely on external power supplies, self-powered flexible sensors can directly convert ambient mechanical energy (such as human motion) into electrical signals, enabling an integrated “sensing-and-powering” functionality [4–6]. This innovation markedly enhances device portability, durability, and user comfort. However, in practical applications—particularly during dynamic human motion monitoring—self-powered flexible sensors face a fundamental challenge: mechanical mismatch at the device–skin interface. Conventional flexible materials generally exhibit a positive Poisson’s ratio, meaning that when stretched (for example, during joint bending), they contract laterally [7–9]. When sensors are attached to highly mobile joints such as the fingers, elbows, or knees, this property gives rise to a paradox: As the joint curvature increases, the sensor is expected to conform more tightly to the skin to capture reliable signals, yet it instead detaches due to lateral contraction, drastically reducing the effective contact area. This loss of interfacial contact triggers a cascade of detrimental effects [1, 10–12]. For sensors relying on contact electrification or piezoresistive mechanisms, signal amplitude rapidly decays or even disappears, leading to data distortion or loss. Simultaneously, the discontinuity in mechanical energy input severely compromises energy harvesting efficiency, undermining the stability of self-powered operation. Consequently, interfacial mechanical mismatch has become the critical bottleneck restricting the precision and reliability of flexible self-powered sensors in realistic dynamic environments. Engineering strain-adaptive, self-powered sensing materials capable of conforming to complex deformation scenarios therefore represents an essential pathway toward overcoming these limitations and achieving next-generation wearable electronics [13–15].

Metamaterials represent engineered systems that exhibit extraordinary physical properties absent in natural materials. Mechanical metamaterials characterized by negative parameters, such as a negative Poisson’s ratio (NPR)—exemplify this paradigm [16–19]. NPR materials display counterintuitive deformation behavior: They contract laterally under axial compression and expand laterally under axial tension. This scale-independent characteristic arises not from the intrinsic chemistry of the constituent material but from the rationally designed geometry of the substructures, manifesting across both macroscopic systems and microscopic unit cells [20–23]. Nature provides vivid inspirations for such bioinspired architectures. Many organisms have evolved structural configurations exhibiting auxetic behavior to adapt to their living environments. For instance, the lacewing wing consists of irregular polygons with re-entrant (concave) angles, which give rise to its negative Poisson’s ratio effect. During wing flexion, the lacewing wing tends to form an arched or dome-like surface whose curvature center aligns with the bending direction. This transverse expansion increases the local convexity of the cross section, significantly enhancing bending stiffness and structural stability—an emergent macroscopic behavior rooted in the microstructural organization of the wing [24–27]. Structurally, the lacewing wing is a composite material composed of a double-layered thin membrane enclosing a vein network. The key to its auxetic nature lies in the widespread presence of re-entrant geometries within this venous lattice. Inspired by this natural design, we constructed a re-entrant hexagonal architecture with multiple concave angles to achieve a negative Poisson’s ratio [28–31]. Under uniaxial tensile loading (e.g., along the horizontal axis), the inclined ligaments undergo in-plane rotational unfolding of the re-entrant angles. This kinematic mechanism compels the vertical struts to displace outward, resulting in lateral expansion and a pronounced NPR effect. Crucially, this rotation-dominated deformation underpins the intrinsic auxetic response of the re-entrant system. When subjected to complex, nonuniform strain fields—such as buckling or conformal bending—the structure exhibits synergistic mechanical behavior: Emergent negative stiffness enhances adaptability to high-curvature surfaces, while the ultralow effective out-of-plane bending modulus ensures strong interfacial adhesion [32, 33]. Recent cutting-edge studies have empirically validated the critical role of these auxetic attributes in wearable electronics. For instance, Kim et al. demonstrated that auxetic-structured reinforcement can effectively eliminate the deformation mismatch between rigid devices and skin by enabling omnidirectional stretchability, thereby preventing delamination [21]. Similarly, Oh et al. [34] utilized the unique shape-fitting capability of auxetic knot architectures to achieve superior conformability on complex body contours for haptic interfaces. Furthermore, Zhou et al. [35] revealed that the synclastic bending behavior of auxetic structures can transform bending deformation into efficient in-plane stretching, amplifying the output performance of energy harvesters by over eightfold. These findings underscore that NPR architectures are not merely structural novelties but are essential for resolving the mechanical and efficiency bottlenecks of current wearable sensors [12, 36–40].

Here, inspired by the lacewing’s natural auxetic structure, we propose a conformal and self-adaptive deformable triboelectric nanogenerator (Auxetic-TENG) that synergistically integrates unique energy conversion and strain-responsive modulation. This capability arises from a flexible negative Poisson’s ratio metastructure, constructed from re-entrant hexagonal cells bridged by triangular ligaments and periodically interconnected into a continuous lattice. Serving as an auxiliary skeletal framework, the NPR metastructure is hierarchically integrated with a PEI-modified collagen positive triboelectric layer and a patterned fluorinated ethylene propylene (FEP) negative triboelectric film, forming a unified functional assembly. The coupled Auxetic-TENG design substantially enhances the performance of dynamic wearable sensing, particularly in joint motion monitoring, by improving both power density and signal fidelity. Furthermore, we quantitatively evaluated the energy conversion efficiency of this bioinspired, structurally tuned self-powered sensor. Comparative experiments verified that the performance optimization originates from the NPR-enabled architecture. To comprehensively assess device performance, we employed a convolutional neural network (CNN)-based deep learning predictive model, integrating experimental measurements and finite element simulations to evaluate power generation efficiency and motion detection sensitivity. Both experimental and computational results consistently confirm that the auxetic effect imparted by the NPR structure significantly strengthens the overall functionality and sensing precision of the device.

## Experimental Section

### Materials and Reagents

Internally synthesized collagen aggregates were prepared. Branched polyethyleneimine (PEI) and fluorinated ethylene propylene (FEP) were purchased from Shanghai Aladdin Biochemical Technology Co., Ltd. (China). Polydimethylsiloxane (PDMS), comprising both base and curing agent components, was obtained from Jiangsu Xianfeng Nano Technology Co., Ltd. Conductive silver paste was sourced from Shanren Advanced Materials Co., Ltd.

### Preparation of CA/PEI

For the fabrication of composite membranes, collagen aggregates (25 wt%) were first dispersed in hexafluoroisopropanol (HFIP) and heated at 45 °C in an oil bath for 2 h until complete dissolution. Separately, polyethyleneimine (PEI, 3 wt%) and triglycidyl isocyanurate (TGIC, 1 wt%) were dissolved in HFIP. The TGIC solution was added dropwise to the collagen acetate (CA) solution under continuous mechanical stirring at 45 °C. After 2 h of reaction, the PEI solution was introduced, and the mixture was further reacted for 6 h. Subsequently, the reaction mixture was dialyzed against deionized water to remove unreacted residues. The resulting homogeneous solution was then cast into a pre-silanized glass mold and cured to obtain CA/PEI composite films with a uniform thickness of 0.3 mm.

### Parameters of COMSOL Simulation for Auxetic-TENG

To simulate realistic auxetic and non-auxetic structures, three-dimensional models were constructed with dimensions of 50 mm (length, x-axis), 40 mm (width, y-axis), and 2 mm (thickness, z-axis). A custom material model was employed, characterized by a density ρ = 1.221 g cm^−3^, Young’s modulus E = 14 MPa, and initial Poisson’s ratios of ν_1_ = − 0.7 (auxetic) and ν_2_ = 0.3 (non-auxetic). To investigate mechanical response distributions during uniaxial tension under lateral–axial co-directional bending, asymmetric boundary loads were applied bilaterally along the x-axis.

### Construction of Auxetic-TENG

The Auxetic-TENG device was assembled using two triboelectric materials: fluorinated ethylene propylene (FEP) and a collagen aggregates/polyethyleneimine (CA/PEI) composite film. Both materials were trimmed to dimensions of 4 cm × 5 cm. A negative-Poisson-ratio elastomer scaffold (thickness: 8 mm) was directly laminated onto the CA/PEI film as the structural framework. An identically patterned FEP layer served as the auxetic compliant backing layer. Silver nanoparticle paste was coated on the reverse side of both the FEP and CA/PEI films to form conductive electrode layers. To ensure controlled separation between the triboelectric layers, two spacer foam sheets (thickness: 0.5 mm each) were inserted between them. An encapsulation layer was then overlaid atop the electrode assembly, functioning as both a substrate and protective surface. Electrical connectivity was established by soldering conductive leads from each electrode layer to external circuitry. The device was hermetically sealed to complete the assembly.

### Characterization of Auxetic-TENG

Surface morphology of the auxetic-structured collagen aggregates/polyethyleneimine (CA/PEI) films was characterized using an optical microscope (Gemini OM, ZEISS, Germany). Chemical bonding analysis was performed via Fourier transform infrared (FTIR) spectroscopy (Tensor II, Bruker, Germany). Functional groups and elemental composition were examined by X-ray photoelectron spectroscopy (XPS, ESCA). Molecular structure was further investigated using a Raman optical microscope. Electrical properties were measured with a Keithley 2635B source meter and a Rigol DS1102E oscilloscope. Data acquisition was managed by a LabVIEW-based system for precise instrument control. Supplementary electrical characterization was conducted using a portable Moku Go device (Liquid Instruments). Pressure application on the triboelectric nanogenerator (TENG) was regulated by a universal testing machine. Numerical simulations employed COMSOL Multiphysics software (Solid Mechanics Module), with physics-controlled meshing (standard size) and unmodified governing equations.

## Results and Discussion

**Fig. 1:**
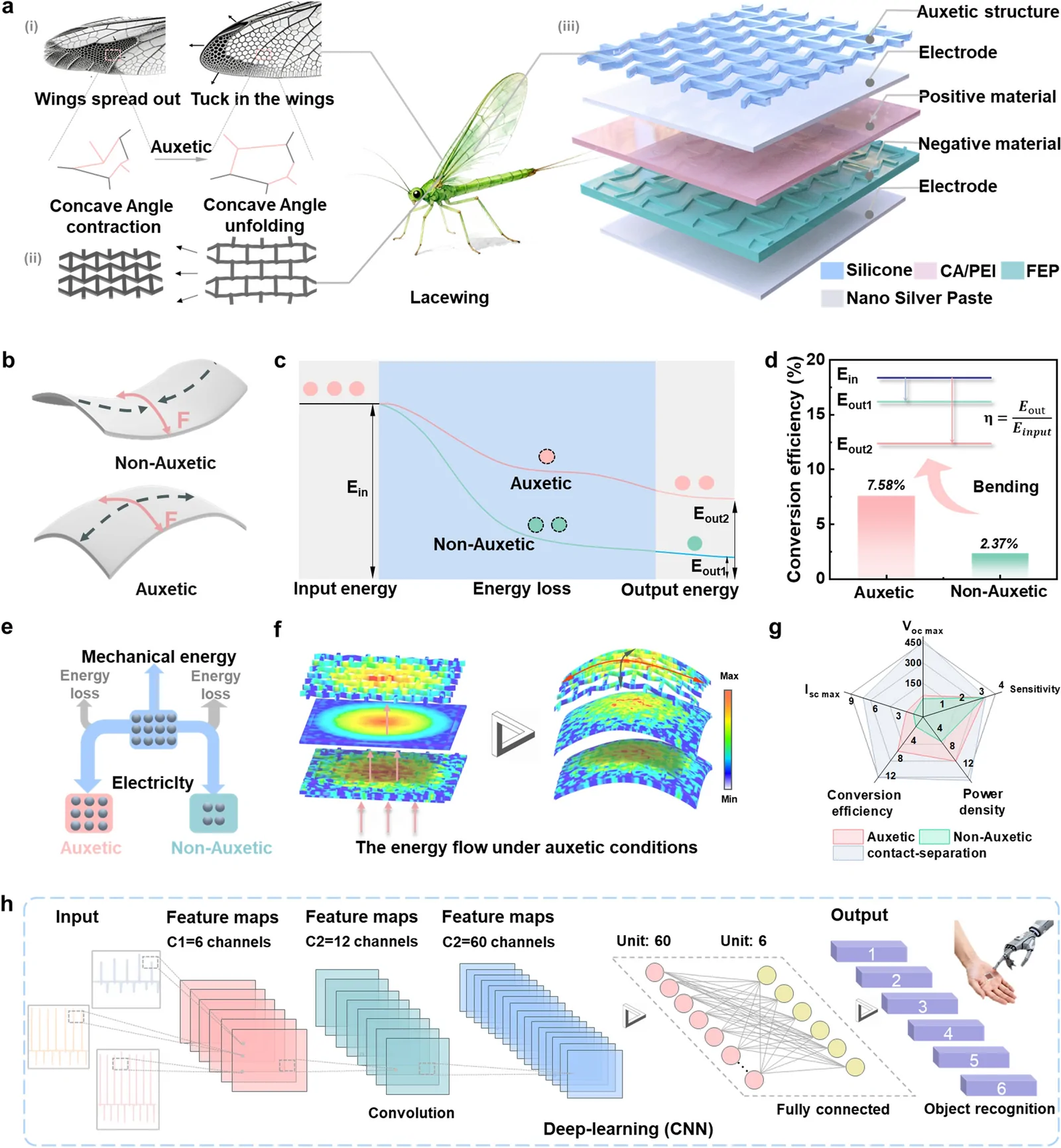
Design, characterization, and applications of the Auxetic-TENG. **a** Macro- and microstructural features of the lacewing wing during folded and extended states (i); structural deformation of the re-entrant hexagonal unit during contraction and expansion (ii); and schematic configuration of the Auxetic-TENG (iii). **b** Comparison of deformation behavior between non-auxetic and auxetic structures under axial bending stress. **c** Energy evolution of non-Auxetic-TENG and Auxetic-TENG at different stages of the energy conversion process. **d, e** Comparison of energy conversion efficiencies of non-Auxetic-TENG and Auxetic-TENG under equivalent mechanical input. **f** Finite element simulation showing the energy (stress) distribution of the Auxetic-TENG under applied load. **g** Performance metrics—including peak voltage, peak current density, energy conversion efficiency, power density, and sensitivity—of non-Auxetic-TENG and Auxetic-TENG in contact–separation and bending modes. **h** CNN-based deep learning model for signal recognition of Auxetic-TENG on mechanical and human palm surfaces

As illustrated in Fig. 1a(i), the fundamental structure of the lacewing wing consists of a lattice framework with numerous re-entrant angles. In its natural folded state, these concave nodes remain contracted; during wing extension, the re-entrant angles unfold, driving the expansion of individual lattice units to significantly enlarge the aerodynamic surface area. Inspired by this biological mechanism, a re-entrant hexagonal metastructure was designed (Fig. 1a(ii)), which exhibits auxetic behavior by expanding laterally in response to axial tension. The detailed device configuration (Fig. 1a(iii)) integrates this auxetic silicone framework with a chemically modified collagen positive layer and a FEP negative layer, utilizing silver nanoparticle electrodes to form the Auxetic-TENG.

The core mechanism governing the performance enhancement is the distinct deformation mode depicted in Fig. 1b. Conventional non-auxetic materials (positive Poisson’s ratio) inherently contract laterally under axial bending, resulting in anticlastic curvature (a saddle shape). This deformation causes the edges of the sensor to curl upward and lift away from the bending surface, leading to a reduction in the effective contact area and interfacial delamination. In stark contrast, the auxetic structure expands laterally under bending stress, inducing synclastic curvature (a dome-like shape). This deformation behavior allows the device to naturally conform to convex surfaces (such as human joints), mechanically regulating the interfacial strain to maintain intimate, gap-free contact. By preventing the formation of voids and slippage at the interface, the auxetic structure ensures that the mechanical energy input is efficiently coupled into the triboelectric layers rather than being dissipated as frictional loss.

This geometric stability directly translates into superior energy harvesting performance. As shown in the energy diagram in Fig. 1c, the auxetic design significantly lowers the energy loss barrier compared to the non-auxetic control, allowing a greater fraction of input mechanical energy (*E*_in_) to be converted into electrical output (*E*_out1_ and *E*_out2_). Consequently, the energy conversion efficiency under bending conditions is enhanced by approximately threefold, increasing from 2.37% in the non-auxetic device to 7.58% in the Auxetic-TENG (Fig. 1d). The schematic in Fig. 1e summarizes this pathway, highlighting how the auxetic framework acts as a mechanical bridge that minimizes loss and maximizes electricity generation.

To further elucidate the energy transfer process and the role of the auxetic architecture under bending deformation, finite element simulations were performed (Fig. 1f). The NPR-enabled film exhibits a more uniform stress distribution under loading. The central collagen layer, which lacks auxetic geometry, experiences an edge-decay effect in stress propagation. Owing to the dual auxetic layers, the Auxetic-TENG demonstrates synchronized bending behavior, thereby confirming its characteristic re-entrant expansion mechanism. A comparative analysis of key performance parameters—including peak voltage, current density, energy conversion efficiency, charge density, and sensitivity—is summarized in Fig. 1g. The Auxetic-TENG outperforms the control device across all metrics in the contact–separation mode. Under bending deformation, its charge density and sensitivity were enhanced to 1.7 and 2.1 times that of the control, respectively, confirming the deformation-induced performance optimization.

To validate the device’s application potential, a CNN-based human–machine interaction recognition model was developed (Fig. 1h). The CNN architecture comprises three convolutional layers, two pooling layers, and two fully connected layers [39–41]. The raw sensor data were preprocessed using short-time Fourier transform (STFT). Eighty percent of the spectrograms were used for training and the remaining twenty percent for testing [42]. After 100 epochs of training, the overall prediction accuracy for all six signal classes exceeded 98%. Comparative analyses of simulated dynamic responses on biological and robotic palm surfaces demonstrate that the Auxetic-TENG offers remarkable advantages in signal fidelity, sensitivity, and environmental adaptability [35, 36, 43–47].

### Characterization and Analysis of Auxetic Films

**Fig. 2:**
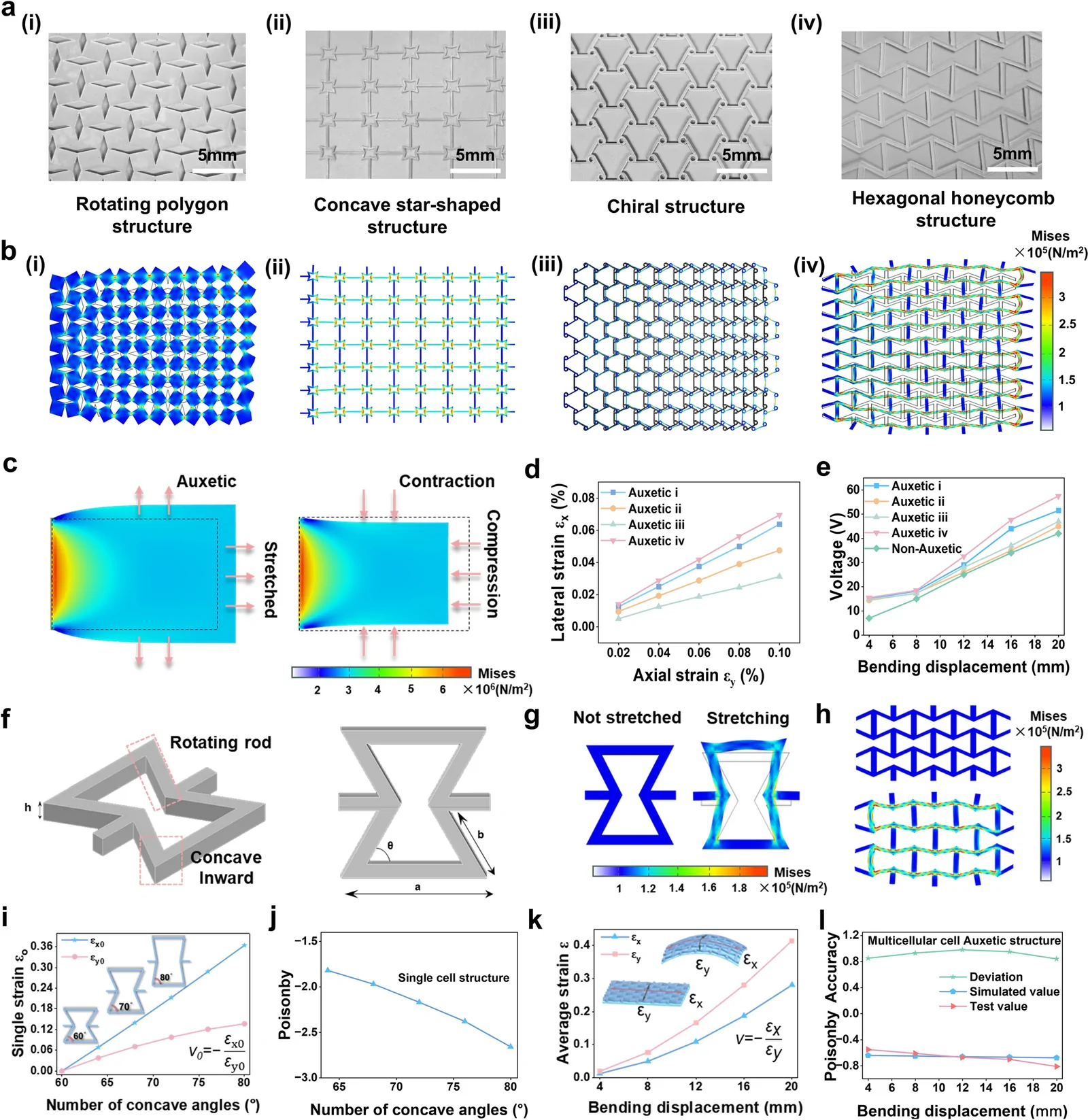
Investigation and Optimization of Auxetic Structures. **a** Optical images of negative Poisson’s ratio films composed of different structural unit cells: rotated polygonal structure (i), star-shaped structure (ii), chiral structure (iii), and re-entrant hexagonal structure (iv). **b** Finite element simulations of the stress distribution in negative Poisson’s ratio films composed of different unit cells: rotated polygonal structure (i), star-shaped structure (ii), chiral structure (iii), and re-entrant hexagonal structure (iv). **c** Finite element simulations of the stress distribution during stretching and compression of the negative Poisson’s ratio structure. **d** Relationship between lateral and axial strains for films composed of four different negative Poisson’s ratio structural unit cells. **e** Output voltage of self-powered sensors based on frameworks composed of four different auxetic structural unit cells and non-auxetic frameworks. **f** 3D conceptual diagram of a single re-entrant hexagonal structural unit. **g** Stress distribution under applied external loads for a single re-entrant hexagonal unit cell in finite element simulations. **h** Stress distribution under applied external loads for multiple re-entrant hexagonal unit cells in a continuous arrangement, as simulated in finite element analysis. **i** Relationship between the concave angle of a single unit cell and the lateral and axial strains of the material. **j** Relationship between the concave angle of a single unit cell and the Poisson’s ratio of the material. **k** Relationship between lateral strain, axial strain, and bending displacement for films composed of multiple re-entrant hexagonal unit cells. **l** Comparison of theoretical and tested Poisson’s ratios for negative Poisson’s ratio films

To elucidate the relationship between the geometric configurations of negative Poisson’s ratio metastructures and their deformation behaviors, this study systematically constructs and analyzes several lattice topologies, as shown in Fig. 2a, including the rotated polygonal structure (i), star-shaped structure (ii), chiral structure (iii), and re-entrant hexagonal structure (iv). All structures exhibit regular periodic geometric features, where the unit angles and hinge connection modes dictate the macroscopic Poisson’s ratio and deformation patterns under external loading. Finite element simulations were performed to model the stress and strain distributions of these structures under uniaxial tensile conditions. Figure 2b illustrates that all four different unit cell-based auxetic films display distinct negative Poisson’s ratio behavior, undergoing lateral expansion during axial stretching. This confirmed that the scale-independent auxetic property originates from the rationally designed substructure geometry rather than inherent material chemistry.

Furthermore, a comparison reveals that the films composed of star-shaped units (ii) and chiral units (iii) show relatively weak auxetic effects under external loads. This is due to the highly symmetric two-dimensional network of the star-shaped structure, where the rotational directions of the unit cells counteract each other. When one node rotates, its neighboring nodes produce an offsetting effect, partially canceling out the lateral expansion, thus weakening the overall NPR effect. In contrast, the chiral structure has circular hinge connections between units, with fixed rotational angles, limiting large rotations and diminishing the macroscopic response. The rotated polygonal structure (i) and re-entrant hexagonal structure (iv), on the other hand, exhibit the most pronounced strain characteristics under stress, with a greater range of hinge rotations leading to significant lateral expansion during stretching.

Figure 2c shows the overall macroscopic deformation of auxetic films under tensile and compressive stress, further demonstrating the advantages of the negative Poisson’s ratio behavior. Figure 2d investigates the relationship between axial and lateral strains for the four different structural unit-based NPR films. The calculation formula is given in Eq. (1):

1 $$\nu = - \frac{{\varepsilon_{x} }}{{\varepsilon_{y} }}$$

where *εₓ* and *ε*_*y*_ represent lateral and axial strains, and *x*, *y* represent the initial lateral and axial lengths, respectively. By comparing the slopes, the differences in Poisson’s ratios are clearly observed. The re-entrant hexagonal (iv) and rotated polygonal (i) structures exhibit the largest absolute values of negative Poisson’s ratio, while the star-shaped (ii) and chiral (iii) structures show weaker effects, consistent with the finite element simulation results described above.

Consequently, the triboelectric output voltage of these films, shown in Fig. 2e, reflects their mechanical properties, with the re-entrant hexagonal structure having the most noticeable impact on the TENG performance. Under bending loads, its auxetic properties are more pronounced, resulting in the highest output voltage. After identifying the most optimal NPR structural framework, we delved into its underlying mechanism. Figure 2f presents a 3D model of a single re-entrant hexagonal unit cell. We observed that the NPR property of the re-entrant hexagonal structure primarily arises from the interaction between its rotation axis and the concave angles. Finite element analysis (Fig. 2g, h) revealed that the strain distribution is concentrated around the concave hinge regions, where the rotational deformation plays a key role in enabling lateral expansion. This localized rotational deformation effectively disperses stress concentrations, allowing the NPR structure to exhibit superior energy dissipation and structural recovery under cyclic deformation.

To verify the simulation results, Fig. 2i presents the relationship between the concave angle of a single unit cell and strain, as calculated and validated using Eqs. (2) and (3):

2 $${\varepsilon }_{y0=}\frac{\mathrm{sin}\theta }{\mathrm{sin}{\theta }_{0}}-1$$

3 $${\varepsilon }_{x0=}\frac{b(\mathrm{cos}{\theta }_{0}-\mathrm{cos}\theta )}{a-b\mathrm{cos}{\theta }_{0}}$$

where *εₓ₀* and *ε*_*y*_*₀* represent the lateral and axial strains of the structure and a, b, *θ*_*0*_,* θ* are the base length, rotation arm length, initial concave angle, and its change, respectively. The formula derivations, leading to Eq. (4), show that as the concave angle increases, the rotating arm induces changes in both lateral and axial strain, resulting in simultaneous lateral and axial expansion. This proves the influence of the concave angle and rotation axis on the NPR structure. Moreover, as calculated from Eq. (4), Fig. 2j shows that the absolute value of the Poisson’s ratio for the re-entrant hexagonal structure increases with the concave angle. This is likely due to the larger rotation angle of the rotation axis as the concave angle increases, which drives the entire structure to unfold, resulting in the auxetic effect.

4 $$\nu = \frac{{b\sin \theta_{0} (\cos \theta_{0} - \cos \theta )}}{{\left( {a - b\cos \theta_{0} } \right)\left( {\sin \theta_{0} - \sin \theta } \right)}}$$

Building on this, we investigated the Poisson’s ratio change of the re-entrant hexagonal film under bending deformation. Due to the mutual interactions between multiple re-entrant hexagonal unit cells, the Poisson’s ratio of the curved film cannot be calculated simply by using a single unit cell’s Poisson’s ratio formula. We performed finite element simulations and equivalent calculations to explore the relationship between bending displacement and Poisson’s ratio in the curved NPR films. The corresponding formulas are given as Eqs. (5) and (6):

5 $$\varepsilon_{x} = \frac{{\sqrt {\left( \frac{d}{2} \right)^{2} + \delta^{2} } - \frac{d}{2}}}{\frac{d}{2}}$$

6 $$\varepsilon_{y} = \frac{{\sqrt {\left( \frac{l}{2} \right)^{2} + \delta^{2} } - \frac{l}{2}}}{\frac{l}{2}}$$

where *d* and *l* represent the lateral and axial lengths of the film and *δ* represents the bending displacement of the film. A comparison of the calculated and experimentally tested Poisson’s ratio values, shown in Fig. 2k, l, reveals a good agreement, with the Poisson’s ratio ranging from − 6.7 to − 7. This confirms that, under bending loads, the film still exhibits the auxetic behavior, forming an arch-like structure and effectively alleviating interface mismatch issues, thus improving the film’s conformability.

### Analysis of the Triboelectric Performance of Auxetic-TENG

**Fig. 3:**
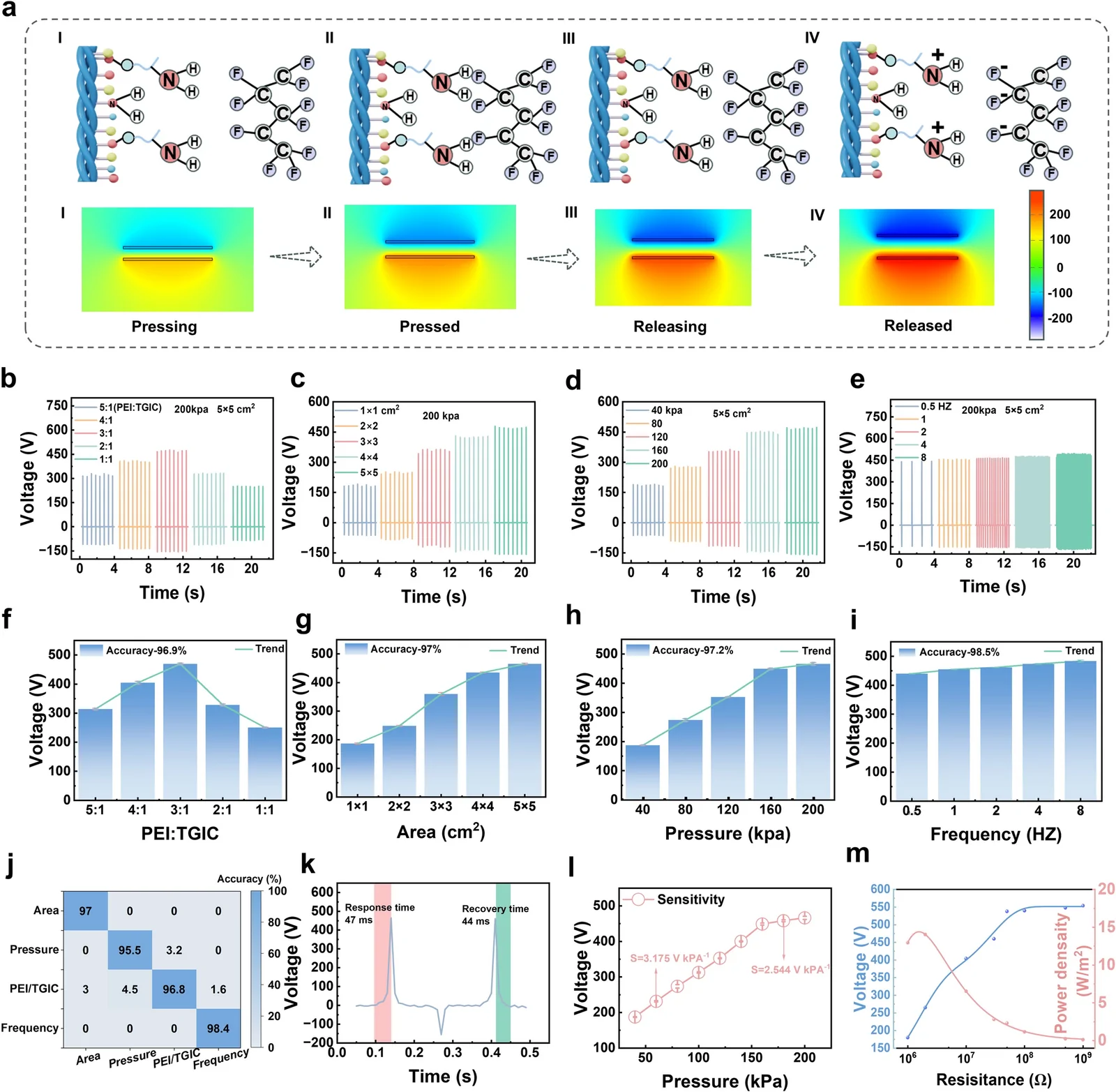
Electrical performance characterization of the Auxetic-TENG. **a** Operational mechanism and corresponding COMSOL simulation of the Auxetic-TENG in contact–separation mode. **b** Output voltage of Auxetic-TENG fabricated with varying PEI/TGIC ratios. **c** Output voltage versus active area of the Auxetic-TENG. **d** Output voltage under different contact pressures. **e** Output voltage at varying contact frequencies. **f** Output voltage trends and consistency of Auxetic-TENG prepared with different PEI/TGIC ratios. **g** Output voltage trends and consistency across varying device areas. **h** Output voltage trends and consistency under different contact pressures. **i** Output voltage trends and consistency at different contact frequencies. **j** Machine learning prediction accuracy versus experimental output signals for varying areas, pressures, PEI/TGIC ratios, and frequencies. **k** Response and recovery times of the Auxetic-TENG. **l** Pressure sensitivity of the Auxetic-TENG. **m** Output voltage and power density versus external load resistance

To investigate the fundamental triboelectric properties of the Auxetic-TENG, its operating mechanism was elucidated through simulation (Fig. 3a and Video S1). When the positive triboelectric layer (CA/PEI) contacts the negative triboelectric layer (FEP), amino functional groups (–NH₂) in PEI establish intimate contact with fluorine functional groups (–F) in FEP. Due to the extreme electronegativity of fluorine, electrons transfer from the amino groups to fluorine atoms. This results in significant negative charge accumulation (negative polarity) on the FEP surface, while the CA/PEI surface acquires positive polarity due to electron depletion. Following separation, this imbalanced charge distribution generates a substantial potential difference between the triboelectric layers. To equilibrate this potential difference, free electrons flow through the external circuit from the negatively charged FEP layer to the positively charged CA/PEI layer, producing characteristic voltage and current pulses. The inherently high impedance of TENGs enables the generation of high-amplitude open-circuit voltage signals—a property directly manifested as ultrahigh sensitivity in sensing applications.

Triboelectric signal output exhibits multifactorial dependency, as systematically investigated through controlled parameter variations. The PEI: TGIC mass ratio fundamentally governs performance characteristics, with Fig. 3b demonstrating peak output signals at the 3:1 ratio. This optimum is further corroborated by Fig. 3f, where signal accuracy reaches 96.9% while revealing diminishing enhancement returns beyond this ratio, consistent with prior material characterization and confirming its superiority. Effective contact area substantially influences output voltage (Fig. 3c), where expansion from 1 × 1 to 5 × 5 cm^2^ elevates open-circuit voltage to 468 V through enhanced total surface charge transfer. The area–voltage relationship (Fig. 3g) exhibits nonlinear progression, with growth rate attenuation leading to saturation as inherent charge transfer limits prevent further accumulation beyond surface charge density thresholds. Notably, 97% signal accuracy persists across all tested areas, confirming output stability. Contact pressure effects (Fig. 3d) show maximum output at 200 kPa across five pressure gradients (40–200 kPa), attributable to improved interfacial contact and charge confinement. The pressure–voltage profile (Fig. 3h) validates this relationship while demonstrating a plateau effect—diminishing returns emerge post-charge saturation due to constrained material deformability, yet maintaining 97.2% signal accuracy. Driving frequency modulation (Fig. 3e, i) reveals that higher frequencies reduce per-cycle charge transfer duration, increasing average current density with 98.5% accuracy. However, potential instability at extreme frequencies establishes intermediate ranges as the optimal operational window. Validation via an CNN-based confusion matrix (Fig. 3j) achieves 97% classification accuracy across signals generated under combinatorial parameter variations—robustly confirming output distinctiveness, repeatability, and stability for precision sensing. Dynamic response assessment (Fig. 3k) demonstrates exceptional sensitivity with 47 ms response/recovery times. Pressure sensitivity profiling (Fig. 3l) yields 3.175 V kPa^−1^ in low-pressure regimes, stabilizing at 2.544 V kPa^−1^ beyond ~ 160 kPa—consistent with charge accumulation limits observed in area and pressure effects. Load-dependent analysis (Fig. 3m) shows maximum energy density (17 W m^−2^) with peak voltage at 1.5 MΩ load resistance, indicating 1.5 MΩ internal impedance. These integrated results substantiate the Auxetic-TENG’s dual functionality in efficient energy harvesting and high-fidelity sensing applications.

### Characterization and Optimization of Mechanical Properties of Auxetic Structures

**Fig. 4:**
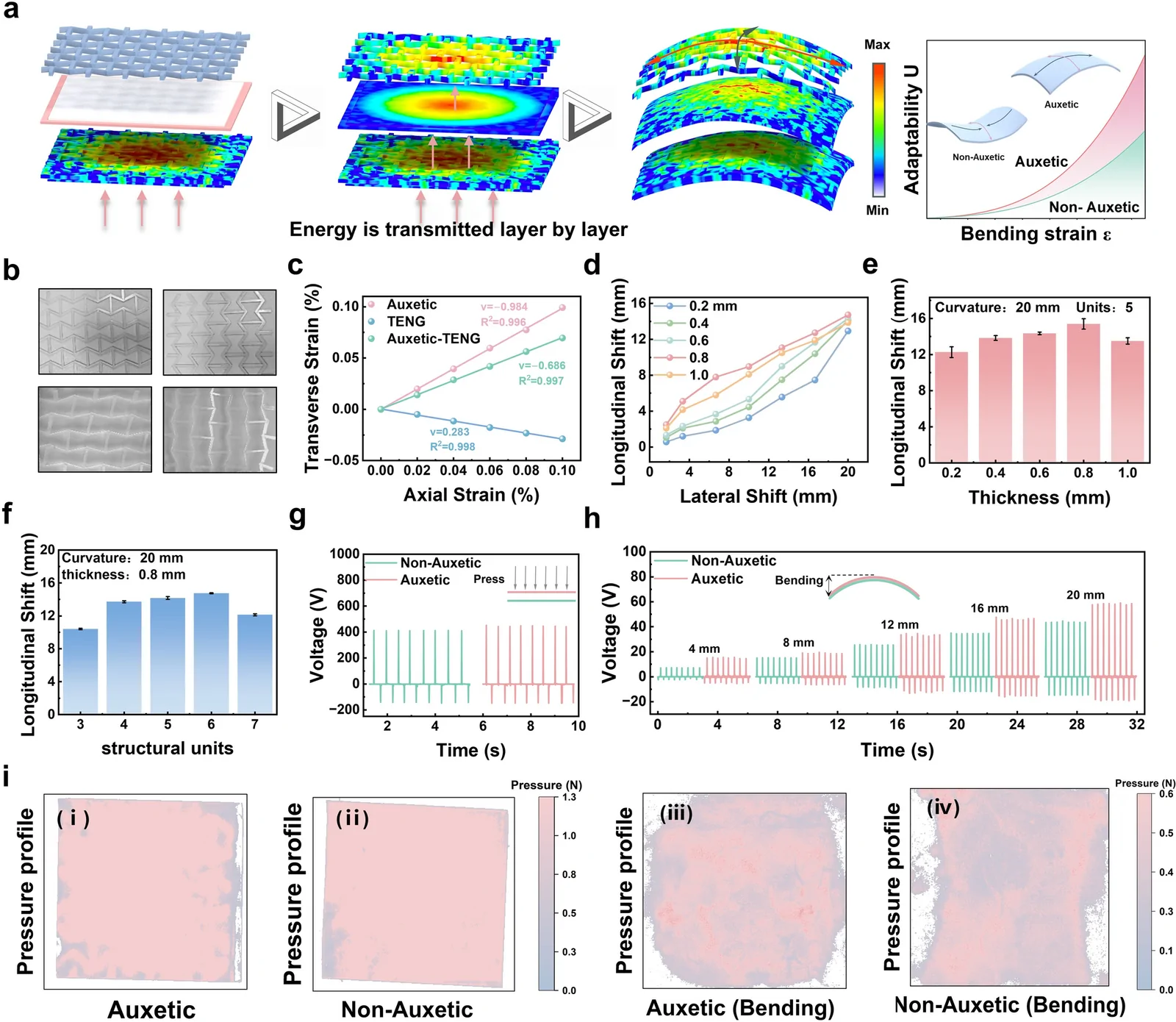
Comparative characterization of auxetic versus non-auxetic structures. **a **Finite element simulations in COMSOL reveal the stress transfer process of the Auxetic-TENG under external loading and compare the strain energy distribution between the auxetic and non-auxetic structures. **b** Photographic representation of the fabricated auxetic structure. **c** Poisson’s ratio measurements for three material systems: auxetic elastomer, CA/PEI composite, and Auxetic-CA/PEI ($\nu = - \varepsilon_{x} /\varepsilon_{y}$). **d** Effect of auxetic material thickness on longitudinal strain under transverse bending strain. **e** Longitudinal strain in Auxetic-CA/PEI at 20 mm transverse bending displacement across thicknesses (0.2, 0.4, 0.6, 0.8, and 1.0 mm). **f** The longitudinal strain of Auxetic-CA/PEI with varying unit cell numbers (3–7) at 20 mm transverse bending displacement. **g** Output signal comparison between non-Auxetic-TENG and Auxetic-TENG in contact–separation mode. **h** Output signal comparison under bending displacements (4, 8, 12, 16, and 20 mm). **i** stress distribution analysis: (i-ii) contact–separation mode, (iii-iv) bending mode

Finite element simulations (Fig. 4a) provide detailed insight into the stress transfer process under external loading. When the center is subjected to force, the auxetic material exhibits a more uniform stress distribution. The re-entrant framework induces an in-plane expansion effect across the entire sensor, thereby increasing the effective contact area and sensitivity, which leads to enhanced triboelectric output and energy conversion efficiency. The physical deformation process shown in Fig. 4b further verifies the structural response, where lateral expansion accompanies longitudinal stretching (structural dimensions shown in Fig. S1). Quantitative Poisson’s ratio measurements (Fig. 4c) provide critical insight into the mechanical interplay of the system. The pure auxetic elastomer exhibits an intrinsic high negative Poisson’s ratio of − 0.98, representing the structural limit of the re-entrant design. In comparison, the fully integrated Auxetic-TENG retains a robust negative Poisson’s ratio of − 0.69. Although the addition of functional layers causes a slight reduction in the auxetic effect compared to the pure framework, the composite device stands in stark contrast with the conventional TENG, which displays a typical positive Poisson’s ratio of + 0.28. This comparison conclusively proves that the auxetic framework acts as the dominant mechanical driver, successfully imparting its strain-adaptive characteristics to the composite system and overriding the inherent positive Poisson’s ratio of the constituent films. Analysis of longitudinal displacement under lateral bending (Fig. 4d, e) shows that the plastomer with thicknesses ranging from 0.2 to 1.0 mm achieves a maximum displacement of 0.8 mm. This optimal behavior originates from the enhanced traction on the CA/PEI film. Structural optimization, as illustrated in Fig. 4f, reveals that the longitudinal deformation generally follows an upward trend as the number of structural units increases. However, once the configuration reaches a critical threshold, the benefit of additional units is offset by heightened intercellular constraints. Specifically, in thicker or overly complex structures (e.g., exceeding 0.8 mm or four units), these constraints suppress the orthogonal strain, ultimately resulting in a reduction of net displacement. Consequently, the 0.8 mm thickness and four-unit configuration represent the peak efficiency point before these inhibitory effects take over.

Electrical output comparisons indicate that, under contact–separation mode, the performance enhancement of the Auxetic-TENG is limited (Fig. 4g). However, during bending deformation (Fig. 4h), the output of the Auxetic-TENG increases sharply with displacement (0–20 mm), exhibiting a 2.8-fold improvement over the non-auxetic configuration. This enhancement arises from the amplified in-plane strain generated during deformation, further corroborated by current output comparisons in Fig. S2. Pressure-mapping analysis (Fig. 4i) elucidates the contact mechanism: Under contact–separation mode, both structures display uniform pressure distributions, whereas during bending, the auxetic structure forms a biaxial expansion pressure field, while the conventional structure exhibits uniaxial stretching accompanied by lateral contraction. This fundamental contrast demonstrates how the auxetic framework optimizes dynamic contact behavior, thereby enhancing sensing performance.

### Self-Power Supply Performance Optimization and Calculation of Self-Powered Sensors

**Fig. 5:**
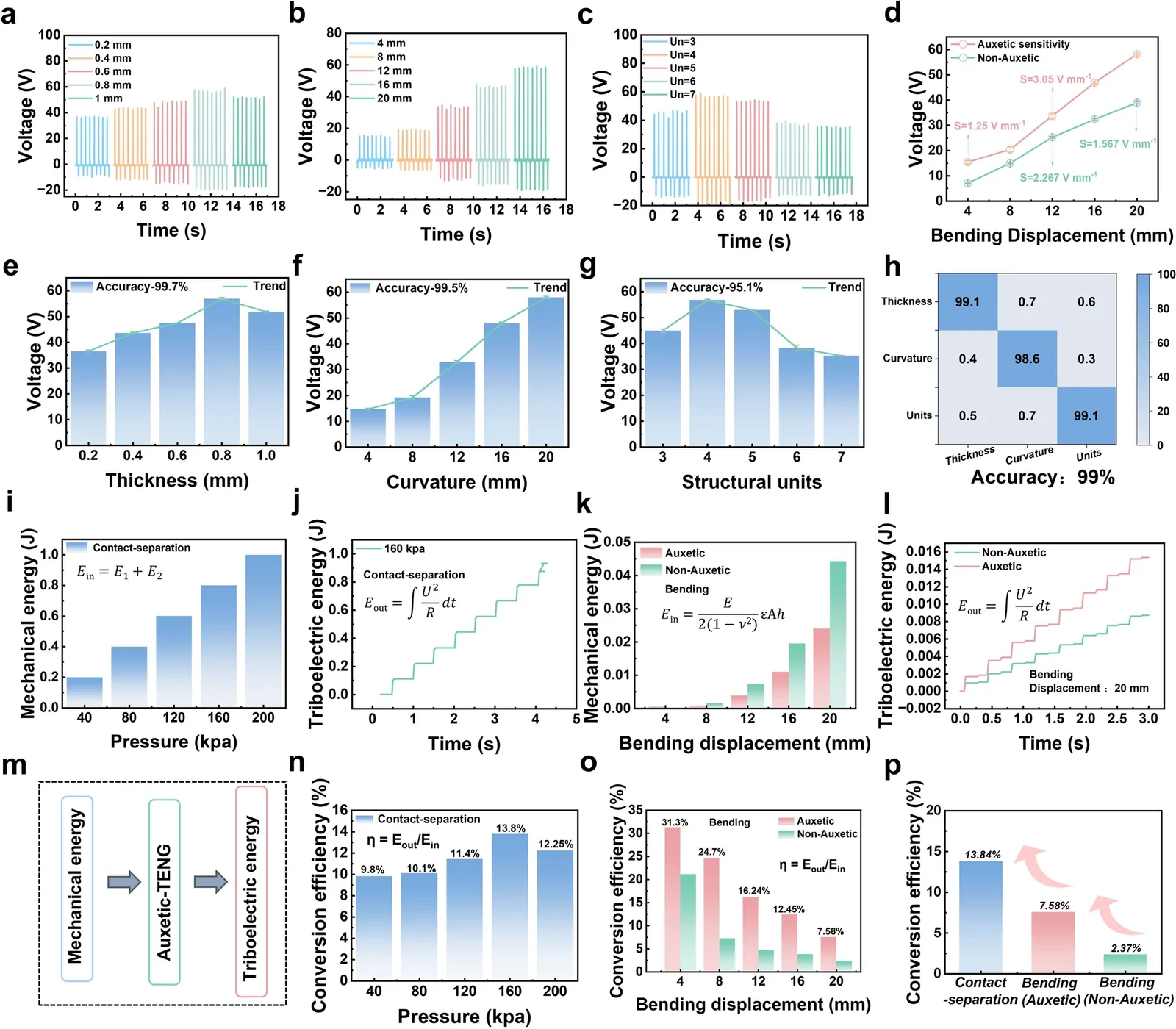
Output optimization and energy conversion efficiency of the Auxetic-TENG. **a** Output voltage versus thickness (0.2–1.0 mm). **b** Output voltage versus bending displacement (4–20 mm). **c** Output voltage versus unit cell number (3–7). **d** Sensitivity comparison of non-Auxetic- and Auxetic-TENG under bending deformation. **e–g** Output signal trends and consistency across: **e** Thickness, **f** Bending displacement, **g** Unit cell variations. **h** Machine learning prediction accuracy for thickness, displacement, and unit cell parameters versus experimental data. **i** Mechanical energy input under contact–separation mode at varying pressures. **j** Triboelectric energy output at 160 kPa (contact–separation mode). **k** Mechanical energy input comparison for non-Auxetic- and Auxetic-TENG under bending displacement. **l** Triboelectric energy output comparison in bending mode. **m** Energy conversion mechanism. **n** Energy conversion efficiency versus pressure (contact–separation mode). **o** Efficiency comparison between non-Auxetic- and Auxetic-TENG in bending mode. **p** Comparative efficiency across operating modes

The thickness-dependent electrical response of the Auxetic-TENG (0.5–1.0 mm) is characterized in Fig. 5a. Peak output voltage (58 V) occurs at 0.8 mm thickness, correlating with maximal longitudinal displacement in mechanical testing (Fig. 4d). This correspondence confirms robust strain transfer–electrical response coupling while achieving 99.7% signal fidelity. Signal stability at this optimized thickness demonstrates 99% output consistency over 100 operational cycles (Fig. 5e). Bending displacement dependence (0–20 mm, Fig. 5b) reveals monotonic voltage enhancement culminating in maximum signal intensity at 20 mm displacement (99.5% accuracy). Figure 5f reinforces this trend through positive voltage–displacement correlation, confirming that large-curvature deformations fully activate auxetic expansion to extend effective contact area. Unit-cell count modulation (3–7 units, Fig. 5c) demonstrates that the 4 × 4 configuration elevates output voltage to 2.3 times baseline values. Quantitative response profiles (Fig. 5g) show < 5% signal fluctuation coefficient and 95.1% output accuracy for this architecture, indicating enhanced signal robustness through structural optimization. Sensitivity analysis under bending deformation (Fig. 5d) reveals markedly divergent behaviors: The Auxetic-TENG exhibits increasing sensitivity with displacement (peaking at 3.05 V mm^−1^), whereas conventional counterparts display progressive sensitivity reduction. This inversion originates from anti-expansion effects in traditional materials during large deformations, consistent with prior observations. Multiparameter optimization was validated via a CNN model (Fig. 5h) analyzing thickness–displacement–unit cell interactions. Following 100 training iterations (80% training/20% validation, 32-neuron architecture), predictions achieved 99% experimental concordance (MSE < 0.1), verifying exceptional signal reliability across operational parameters.

Energy conversion efficiency was quantified across operational modes (Fig. 5i). In contact–separation mode, mechanical energy derives primarily from pressure work (*W*ₚ) and gravitational potential variation (*ΔE*_g_), with total mechanical energy (*E*_in_) expressed as (full derivation in Tables S1–S3):

7 $$E_{{{\mathrm{in}}}} = W_{{\mathrm{p}}} + \Delta E_{{\mathrm{g}}}$$

At constant Δ*E*_g_, pressure intensity dominantly affects *E*ₘ. Increasing pressure from 40 to 200 kPa elevates *E*ₘ to 1 J maximum. Instantaneous triboelectric energy (*E*ₜ) reached 0.1105 J under optimal conditions (Fig. 5j).

8 $${E}_{\mathrm{out}}\,=\,\int \frac{{U}^{2}}{R}{\mathrm{d}{t}}$$

Conversion efficiency peaked at 13.8% under 160 kPa pressure (Fig. 5n), calculated via:

9 $$\eta = \frac{{E_{{{\mathrm{out}}}} }}{{E_{{{\mathrm{in}}}} }}$$

In bending mode (Fig. 5k), biaxial strain energy calculations show Auxetic-TENG achieves maximum mechanical energy (0.024 J) at 20 mm displacement versus 0.044 J for non-auxetic devices (Fig. S3). The specific calculation formula is as follows:

10 $$E_{{{\mathrm{in}}}} = \frac{E}{{2(1{ - }\nu^{2} )}}\varepsilon Ah$$

11 $$\varepsilon = \varepsilon_{x}^{2} + \varepsilon_{y}^{2} + 2\nu \varepsilon_{x} \varepsilon_{y}$$

Among them, *E* represents the elastic modulus of the material (Fig. S4), *v* is the Poisson’s ratio of the material, *ε* is the strain magnitude of the material, *ε*_*x*_ is the transverse strain, and *ε*_*y*_ is the longitudinal strain, for further details, please refer to Note S4. A represents the area of the material and h represents the thickness of the material. Corresponding triboelectric energies were 0.00182 J (auxetic) and 0.00105 J (non-auxetic) (Figs. 5l and S5), yielding 7.58% versus 2.37% conversion efficiency—a 3.2-fold enhancement for the auxetic structure (Fig. 5o). Energy conversion mechanisms (Figs. 5m and S6) demonstrate how mechanical energy transforms into triboelectric energy (manifested as sensor signals or LED illumination) in both operational modes. Critical efficiency comparisons (Fig. 5p) reveal that contact–separation mode achieves peak efficiency due to minimal contact area loss auxetic structures exhibit 3.1 higher bending-mode efficiency than conventional counterparts. This enhancement stems from auxetic-driven contact area preservation, whereas conventional materials suffer significant area loss from strain mismatch during bending.

### Self-Power Supply Performance of Self-Powered Sensors

Figure S7 characterizes the capacitor charging performance of the Auxetic-TENG across capacitances (4.7, 10, 22, 33, and 47 μF). The device achieves saturation voltage for a 4.7 μF capacitor within 4 s, while maintaining stable charging control (~ 60 s) even for the 47 μF capacitor. Frequency-dependent analysis (0.5–8 Hz) further reveals accelerated charging rates and optimized energy conversion efficiency at 8 Hz under equivalent capacitance conditions. Practical application validation was demonstrated through electronic timer activation (Fig. S8). Continuous operation for 40 s successfully powered the timer, with stable charge–discharge cycles confirming reliable self-powering functionality. Drive capacity testing (Fig. S9) verified the capability to simultaneously illuminate 47 commercial electroluminescent bulbs. Long-term operational stability was assessed over a 50-min continuous test (Fig. S10). Both output voltage and current signals remained highly stable throughout the extended duration, confirming reliability for sustained energy harvesting and sensing applications. These collective results substantiate the practical utility of this self-powered sensor system.

### Sensing Applications of Self-Powered Sensors

**Fig. 6:**
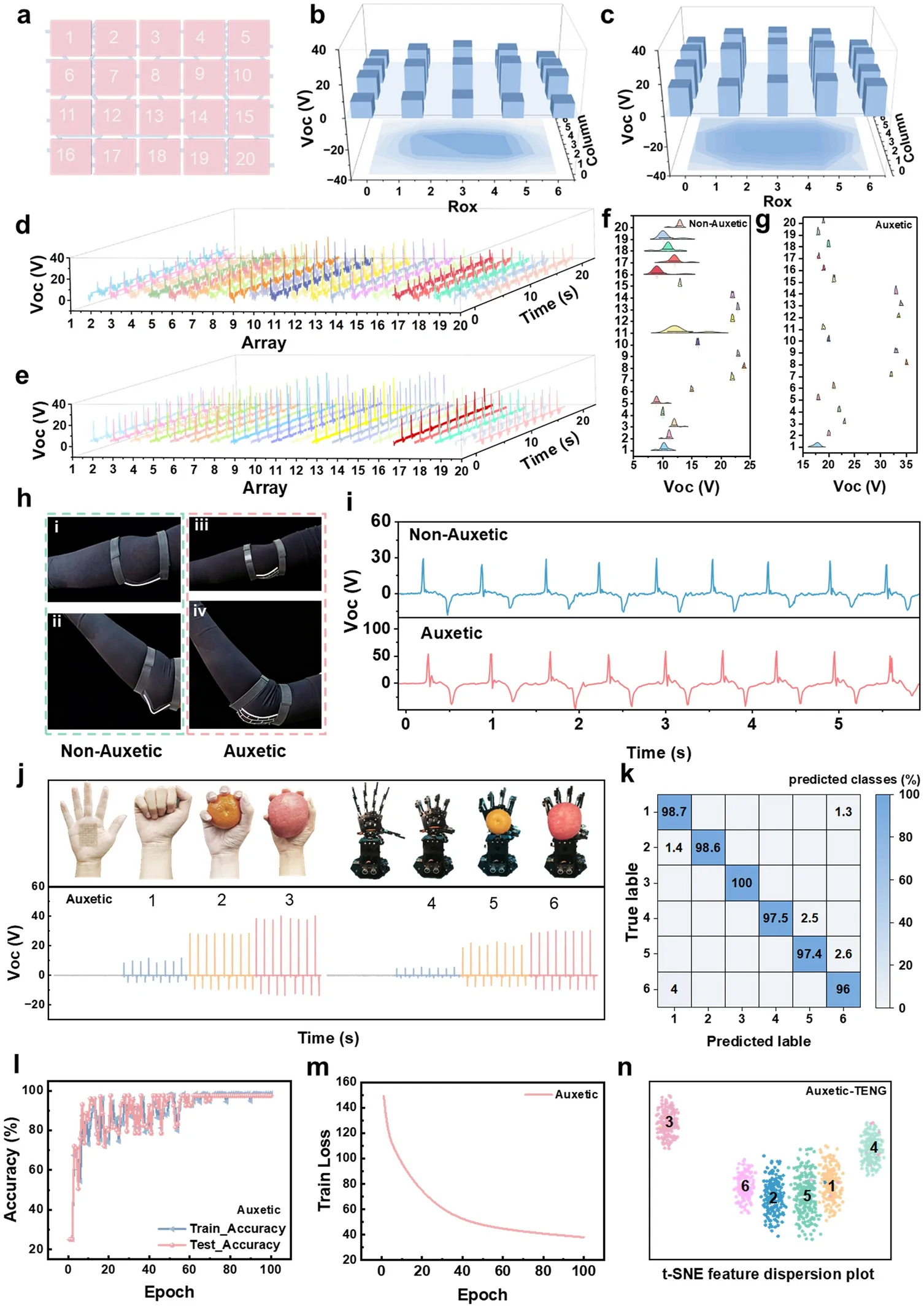
Sensing applications of the Auxetic-TENG. **a** Schematic of a 20-element array with auxetic structures. Spatial signal distribution when contacting objects: **b** Non-Auxetic-TENG, **c** Auxetic-TENG affixed to palmar surface. Corresponding stable sensing outputs: **d** Non-Auxetic-TENG, **e** Auxetic-TENG. Statistical distribution analysis (raincloud plots) of the peak voltage values for the **f** Non-Auxetic- and **g** Auxetic-TENG. **h** Optical photographs comparing the contact status of the devices attached to a human elbow: (i, ii) the non-auxetic control exhibiting edge delamination and (iii, iv) the Auxetic-TENG maintaining conformal contact under flexion. **i** The corresponding open-circuit voltage output generated under a 90° elbow bending angle for the non-Auxetic-TENG and Auxetic-TENG, highlighting the significant signal enhancement achieved by the auxetic structure. **j** Application in intelligent grasping: Photographs of human and robotic hands grasping different objects (labeled 1–6) alongside their corresponding generated voltage signal patterns. **k** Confusion matrix of the CNN-based machine learning model, revealing a high classification accuracy for object recognition. **l** Training and testing accuracy curves of the CNN model over 100 epochs. **m** Training loss convergence curve. **n** t-SNE visualization of the high-dimensional feature distribution, demonstrating clear clustering and separation for the six different grasping categories

To evaluate the scalability and practical versatility of the proposed design, a pixelated sensor array consisting of 4 × 5 sensing units was fabricated (Fig. 6a). A comparative study was conducted to assess the electrical uniformity and mechanical stability between the auxetic and non-auxetic configurations. As visualized in the 3D spatial mapping of open-circuit voltage, the non-auxetic array exhibited a nonuniform distribution with notable signal degradation at the peripheral units (Fig. 6b). In stark contrast, the auxetic array demonstrated superior output consistency across all 20 channels, maintaining a uniform potential distribution (Fig. 6c). This enhancement is attributed to the synclastic curvature of the auxetic framework, which effectively mitigates the edge delamination issues prevalent in conventional films.

The dynamic signal stability was further scrutinized through continuous operation over 20 s. The 3D waterfall plots reveal that the non-auxetic sensor suffered from significant baseline fluctuations and pronounced noise, indicative of unstable contact (Fig. 6d). Conversely, the Auxetic-TENG generated robust, high-fidelity signals with a stable baseline and minimal attenuation (Fig. 6e). To quantitatively validate this stability, a statistical raincloud plot analysis was performed on the peak voltage distribution. The auxetic group (Fig. 6g) exhibited a markedly narrower distribution with centered peak values compared to the dispersed distribution of the non-auxetic control (Fig. 6f), confirming its exceptional repeatability and mechanical reliability. To further substantiate the device’s robustness under repeated deformation, long-term durability tests were performed. The auxetic structure displayed stable mechanical hysteresis behavior after 50 tensile cycles (Fig. S11a) and maintained consistent electrical output voltage over 1000 continuous bending cycles (Fig. S11b), ensuring prolonged operational integrity.

The conformability advantage was experimentally verified on a human elbow under varying degrees of flexion. While the non-auxetic device showed visible detachment and edge curling at large bending angles (Fig. 6h(i), (ii)), the Auxetic-TENG maintained seamless, conformal contact throughout the motion (Fig. 6h(iii), (iv)). Consequently, under a 90° bending condition, the Auxetic-TENG delivered a significantly higher and more stable voltage output (Fig. 6i) compared to the erratic signals of the control device, demonstrating its suitability for monitoring complex joint movements.

Leveraging the high signal-to-noise ratio of the auxetic sensor, an intelligent perception system was developed for object recognition. A convolutional neural network (CNN) was employed to classify signal patterns generated by grasping six distinct objects (labeled 1–6), including bare hands, fruits, and robotic grippers (Fig. 6j). The dataset was processed using the CNN architecture detailed in Fig. S18. The model achieved rapid convergence, as evidenced by the accuracy trajectory (Fig. 6l) and training loss curve (Fig. 6m), reaching an overall recognition accuracy of over 98%. The confusion matrix (Fig. 6k) confirms precise classification with minimal error rates across all categories. Furthermore, the t-distributed Stochastic Neighbor Embedding (t-SNE) visualization (Fig. 6n) illustrates distinct and well-separated clusters for each grasping scenario, validating the high discriminative power of the Auxetic-TENG sensing system for advanced human–machine interface applications.

## Conclusion

In summary, this work establishes a generalizable design strategy to resolve the intrinsic mechanical mismatch at soft bio-interfaces by integrating bioinspired auxetic metastructures with triboelectric sensing technology. Drawing inspiration from the lacewing wing, we engineered a re-entrant hexagonal architecture that transforms the conventional anticlastic deformation behavior into synclastic curvature. This geometric adaptation fundamentally suppresses edge delamination and enables gap-free, conformal contact with complex 3D joint surfaces, thereby serving as a robust mechanical regulator. Consequently, the proposed Auxetic-TENG achieves remarkable electromechanical stability and efficiency. In contact–separation mode, the device delivers a peak voltage of 478 V and a high mechanical-to-electrical energy conversion efficiency of 13.8%. Crucially, under dynamic bending conditions, the auxetic-induced contact optimization leads to a threefold enhancement in efficiency (7.58%) compared to non-auxetic counterparts. Furthermore, by synergizing this mechanically adaptive hardware with a CNN-based deep learning algorithm, the system demonstrates exceptional capability in intelligent perception, achieving over 99% accuracy in object recognition. Collectively, these results not only demonstrate a high-performance self-powered sensor but also provide a methodological blueprint for designing next-generation wearable electronics that simultaneously achieve mechanical compatibility, energy efficiency, and intelligent sensing.

## Supplementary Information

Below is the link to the electronic supplementary material.

Supplementary file1 (MP4 70 kb)

Supplementary file2 (DOCX 2539 kb)
