# Supplementary material for: Bioinspired Auxetic Metastructures Enable Biomechanically Adaptive, Machine Learning-Enhanced Self-Powered Sensing with Ultrahigh Efficiency
Source: Nanomicro Lett. 2026 Mar 18;18:286. doi: 10.1007/s40820-026-02125-8 (PMC12996474; doi:10.1007/s40820-026-02125-8)
Supplement: Supplementary file 2 — Supplementary file2 (DOCX 2539 kb) [file 40820_2026_2125_MOESM2_ESM.docx]

Supporting Information for

**Bioinspired Auxetic Metastructures Enable Biomechanically Adaptive, Machine Learning-Enhanced Self-Powered Sensing with Ultrahigh Efficiency**

Wei Wang ^1^, Xuechuan Wang ^1,^ *, Linbin Li ^2^, Yi Zhou ^2^, Wenlong Zhang ^1^, Long Xing ^1^, Long Xie ^2^, Yitong Wang ^1^, Ouyang Yue ^1,^ *, Xinhua Liu ^1,^ *

^1^ College of Bioresources Chemical and Materials Engineering, Shaanxi University of Science & Technology, Xi’an 710021, P. R. China

^2^ College of Chemistry and Chemical Engineering, Shaanxi University of Science & Technology, Xi’an 710021, P. R. China

*Corresponding authors. E-mail: [liuxinhua@sust.edu.cn](mailto:liuxinhua@sust.edu.cn) (Xinhua Liu); [ouyangyue@sust.edu.cn](mailto:ouyangyue@sust.edu.cn) (Ouyang Yue); [wangxc@sust.edu.cn](mailto:wangxc@sust.edu.cn) (Xuechuan Wang)

**Note S1 Basic Characterization of CA/PEI Materials**

Validation of successful CA/PEI preparation was conducted through FTIR spectroscopy at varying PEI/TGIC ratios (**Fig. S13**). The broad absorption peak at 3286.6 cm⁻¹ arises from overlapping N-H stretching vibrations of PEI’s secondary amine groups (-NH- at 3284 cm⁻¹) and O-H stretching from hydrolyzed TGIC. Significantly enhanced C-H characteristic peaks at 2929.7 cm⁻¹ and 2846.8 cm⁻¹ originate from the aliphatic chains of PEI. The shift of the amide I band to 1685.7 cm⁻¹ indicates new amide bond (C=O) formation through reactions between TGIC epoxy groups and collagen amino groups. Overlapping signals at 1460.1 cm⁻¹—attributable to the collagen amide II band and PEI methylene bending vibration (1460 cm⁻¹)—provide evidence for PEI network crosslinking. Furthermore, the C-N stretching vibration at 1029.9 cm⁻¹ confirms amine group participation in the reaction. Raman point scans of TGIC and PEI (**Fig. S14a**) reveal distinct peaks at 1457.7 cm⁻¹, 2963.3 cm⁻¹, and 2875.2 cm⁻¹ corresponding to PEI’s aliphatic chain structures (-CH₂- and -CH₃), consistent with its branched polyethylenimine characteristics. The peak at 1257.7 cm⁻¹ represents the C-N stretching vibration of TGIC’s triazine ring, confirming core structural integrity. Analysis of CA/PEI samples (**Fig. S14b**) shows the characteristic crosslinking peak of TGIC’s triazine ring C-N bond at 1286.1 cm⁻¹ decreases in intensity with reduced TGIC content, indicating higher TGIC proportions promote crosslinking. Concurrently, peak intensity in the aliphatic C-H region (2969.1 cm⁻¹) increases with higher PEI content, signifying PEI dominance and insufficient crosslinking. Notably, a distinct and minimally perturbed amide I band peak within 1600-1700 cm⁻r is observed at the 3:1 PEI: TGIC ratio, suggesting this ratio is optimal. XPS analysis further confirmed CA/PEI preparation (**Fig. S15a**), showing significantly enhanced N1s peak intensity for modified CA/PEI versus unmodified CA. Peak deconvolution fitting of core levels revealed a C-N bond at 400.4 eV in N 1s (**Fig. S15b**), alongside C=O (532.0 eV) and C-O (533.8 eV) bonds in O1s (**Fig. S15c**). While confirming amino and carbonyl group presence in CA/PEI, these features alone cannot prove successful PEI crosslinking. Critically, the appearance of C-O-C at 286 eV in C 1s (**Fig. S15d**) provides direct evidence for the TGIC ring-opening reaction and successful PEI introduction onto CA. Water contact angle measurements across PEI: TGIC ratios (**Fig. S16a**) demonstrate increased hydrophilicity with higher free amino group content, where the lowest contact angle at the 3:1 ratio correlates with maximal amino groups and strongest electropositivity. **Fig. S16b** details the significant hydrophilic transformation post-modification.

**Note S2 Detailed Architecture of the 1D-CNN Model**

The Convolutional Neural Network (CNN) architecture, as illustrated in **Fig. S19 and Tables S4-S5**, is explicitly designed to process the 1D time-series triboelectric signals. The network consists of three sequential convolutional blocks followed by two fully connected (dense) layers.

Feature Extraction: The input signal first passes through the first convolutional layer (C1) with 6 filters to capture low-level temporal features. This is followed by a second layer (C2) with 12 filters, and a third layer (C3) with 60 filters, which extracts high-level semantic patterns from the signal.

Classification: The output feature maps from C3 are flattened and fed into a fully connected layer (FC1) containing 60 neurons. The final output layer (FC2) consists of 6 neurons, corresponding to the six target classification categories. A Softmax function is applied to the output to generate the probability distribution for object recognition.

**Note S3 Theoretical Derivation of the Performance Enhancement Mechanism**

This section provides a rigorous theoretical framework to elucidate the physical mechanism by which the auxetic metastructure enhances the triboelectric performance. We establish an analytical model linking the structural mechanics of Poisson’s ratio to the contact electrification dynamics.

1. Mechanics of Synclastic Curvature and Interface Conformability

The fundamental advantage of the auxetic sensor lies in its ability to exhibit synclastic curvature, which minimizes the geometric mismatch with human joints.

Consider a thin elastomeric sheet with thickness $h$ subject to a pure bending moment $M$ along the $x$-axis (longitudinal direction). The longitudinal curvature is defined as $\kappa_{x}=1/R_{x}$. According to the classical plate theory, the transverse curvature $\kappa_{y}$ induced by the Poisson’s effect is governed by the relation:

$\kappa_{y}=-\nu\cdot\kappa_{x}$ (S1)

where $\nu$ is the Poisson’s ratio of the structural material.

For conventional materials (e.g., standard elastomers where $\nu\approx0.5$), $\kappa_{y}$ has the opposite sign to $\kappa_{x}$. If the sheet is bent downwards ($\kappa_{x}>0$), the transverse direction bends upwards ($\kappa_{y}<0$). This results in anticlastic curvature (a saddle shape).

The vertical deviation (gap height) $z(y)$ at the edges relative to the center can be approximated by geometric expansion:

$z(y)\approx\frac{1}{2}\kappa_{y}y^{2}=-\frac{1}{2}\nu\kappa_{x}y^{2}$ (S2)

This negative deviation implies that the edges lift away from the underlying convex surface (e.g., skin), creating a delamination gap $\delta_{gap}$.

For our re-entrant structure with a negative Poisson’s ratio ($\nu<0$), the transverse curvature $\kappa_{y}$ has the same sign as $\kappa_{x}$.

$\kappa_{y}=-(-|\nu|)\cdot\kappa_{x}=|\nu|\cdot\kappa_{x}>0$ (S3)

This results in synclastic curvature (a dome shape). The deformation naturally follows the convex curvature of the joint, minimizing the gap height ($z(y)\to0$). This geometric matching ensures that the interface remains in conformal contact without restoring forces causing detachment.

#### 2. Evolution of Effective Contact Area ($S_{eff}$)

The electrical output of a contact-separation TENG is strictly proportional to the effective contact area $S_{eff}(t)$. We model the effective area as the integral of the contact interface $\Omega$ weighted by a contact pressure distribution function $H(P)$:

$\text{S}_{\text{eff}}\text{(t)=}\iint_{\Omega}^{...} \text{H(P(x,y,t)-}\text{P}_{\text{th}}\text{) dxdy}$ (S4)

where $P(x,y,t)$ is the instantaneous contact pressure and $P_{th}$ is the minimum pressure threshold for effective charge transfer.

For the Non-Auxetic Control: Due to the edge lifting derived in Eq. (S2), the contact region is restricted to a narrow central strip. As the bending curvature $\kappa_{x}$ increases, the effective width $W_{eff}$ decreases:

$W_{eff,non}\approx W_{0}\cdot(1-\beta\cdot\nu_{pos}\cdot\kappa_{x})$ (S5)

where $\beta$ is a geometric factor. Thus, $S_{eff}$ decays rapidly during bending.

For the Auxetic-TENG: The lateral expansion ($\Delta W>0$) inherently induced by the negative Poisson’s ratio counteracts the area reduction. The effective width evolves as:

$W_{eff,aux}\approx W_{0}\cdot(1+|\nu_{neg}|\cdot\varepsilon_{axial})$ (S6)

Consequently, the effective contact area not only maintains its stability but actually increase under tensile bending:

$S_{eff,aux}(t)\geq S_{0}$ (S7)

This derivation theoretically confirms the experimental observation that auxetic structures prevent signal degradation under large deformations.

#### 3. Enhancement of Electrical Output and Current Density

Based on the variable capacitance model of TENG, the short-circuit current ($I_{sc}$) is defined as the rate of charge flow:

$I_{sc}(t)=\frac{dQ}{dt}=\frac{d(\sigma\cdot S_{eff}(t))}{dt}$ (S8)

Expanding the derivative using the chain rule:

$I_{sc}(t)=\sigma\frac{dS_{eff}(t)}{dt}+S_{eff}(t)\frac{d\sigma}{dt}$ (S9)

Assuming a constant surface charge density $\sigma$ in the steady state, the current is dominated by the rate of change of the contact area $\frac{dS_{eff}}{dt}$.

In the Auxetic-TENG, the re-entrant structure undergoes rapid lateral expansion during the bending action. This introduces an additional dynamic term to the area change rate compared to the static non-auxetic counterpart:

$\left( \frac{dS_{eff}}{dt} \right)_{aux}=\frac{dS_{bending}}{dt}+\underset{Auxetic Contribution}{\underbrace{\frac{dS_{lateral expansion}}{dt}}}$ (S10)

Since $\frac{dS_{lateral expansion}}{dt}>0$ for auxetic materials under tension, the magnitude of $I_{sc}$ is significantly amplified. This mathematical relationship explains why the auxetic sensor exhibits higher current peaks and sensitivity during dynamic joint movements.

#### 4. Energy Conversion Efficiency Formulation

The energy conversion efficiency ($\eta$) is the ratio of generated electrical energy ($E_{elec}$) to the input mechanical work ($W_{mech}$):

$\eta=\frac{E_{elec}}{W_{mech}}=\frac{\int V(t)I(t)dt}{\int F(t)d\delta(t)}$ (S11)

The input work $W_{mech}$ consists of the strain energy stored in the material ($U_{strain}$) and the energy dissipated through friction and interfacial slippage ($E_{diss}$):

$W_{mech}=U_{strain}+E_{diss}$ (S12)

Friction Reduction: In non-auxetic devices, the mismatch-induced sliding at the skin-device interface generates significant frictional heat ($E_{diss}\uparrow$).

Optimal Coupling: The auxetic structure’s conformal contact implies that the relative displacement (slip) between the sensor and the skin is minimized ($\Delta u_{slip}\approx0$). Therefore, the dissipative term $E_{diss}\to0$. By minimizing $W_{mech}$ (wasteful dissipation) and maximizing $E_{elec}$ (via enhanced $S_{eff}$), the total efficiency $\eta$ is theoretically maximized.

**Note S4 Theoretical Assumptions and Boundary Conditions for Energy Conversion Efficiency Calculation**

This note provides a detailed clarification of the physical assumptions, boundary conditions, and applicable scope underlying the mechanical energy input and conversion efficiency calculations presented in Section 2.4. To ensure a transparent and standardized evaluation of the device's structural performance, the theoretical derivation relies on specific approximations regarding material behavior and strain distribution.

The first critical assumption is the linear elastic behavior of the composite material. The calculation of elastic strain energy utilizes a constant Young’s modulus, effectively modeling the stress-strain relationship as linear within the operational window. While the constituent polymers, such as PDMS, are inherently hyperelastic, the mechanical deformations in our testing range (bending displacement up to 20 mm) correspond to global effective strains where the material response remains predominantly linear. This first-order approximation allows for an analytical derivation that highlights the fundamental comparative advantages of the auxetic structure without being obscured by the complexities of non-linear finite element modeling.

Furthermore, the theoretical model treats the discrete auxetic lattice as a continuous effective medium with homogenized mechanical properties. Consequently, the strain term in the energy equation represents the volume-averaged equivalent strain rather than localized peak strains at the structural hinges. In the bending mode, although strain gradients exist along the thickness, utilizing classical plate theory to integrate over the effective volume provides a representative energy input value. This homogenization strategy facilitates a direct macroscopic evaluation of the Auxetic Effect, such as the enhancement from synclastic curvature, independent of microscopic stress concentrations.

Finally, it is important to note that the input energy is defined strictly as the stored elastic potential energy, thereby excluding energy dissipated due to material viscoelasticity (hysteresis loops) and air damping. As a result, the reported efficiency represents the Elastic Energy Conversion Efficiency. This metric specifically quantifies the device's capability to convert recoverable mechanical energy into electricity, isolating the electromechanical coupling performance from the material's intrinsic thermal damping. Under the quasi-static or low-frequency actuation conditions employed in this study, where elastic restoring forces dominate over viscous damping forces, this definition serves as a robust and standardized benchmark for assessing the superior structural capability of the Auxetic-TENG.

**Supplementary Figures and Tables**


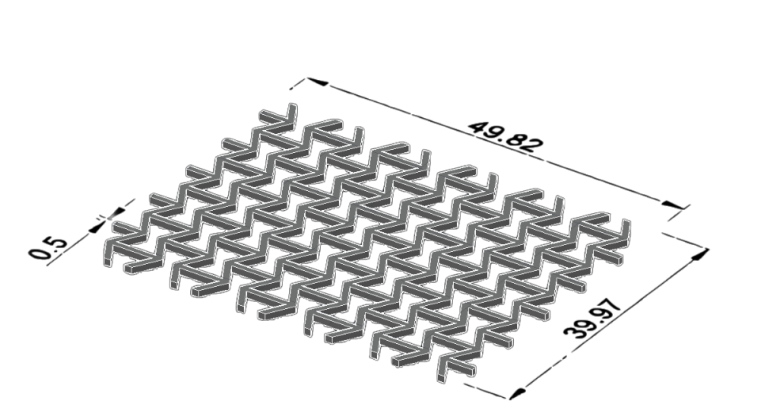

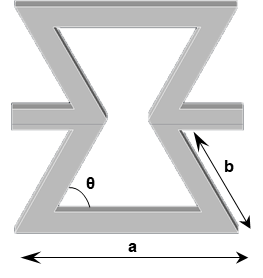


**Fig. S1** **Schematic diagram and dimensions of the auxetic structure [S**1, S2] **here, a=4.93, b=3.53, and θ_0_=60°**


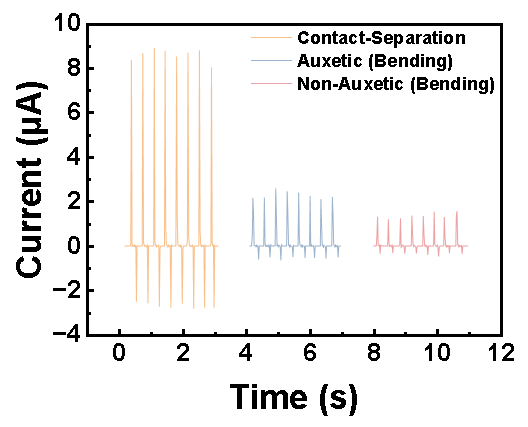


**Fig. S2** **Output current of the Auxetic-TENG in contact-separation mode, and comparison of output currents between Auxetic- and Non-Auxetic-TENGs in bending mode [S**3]


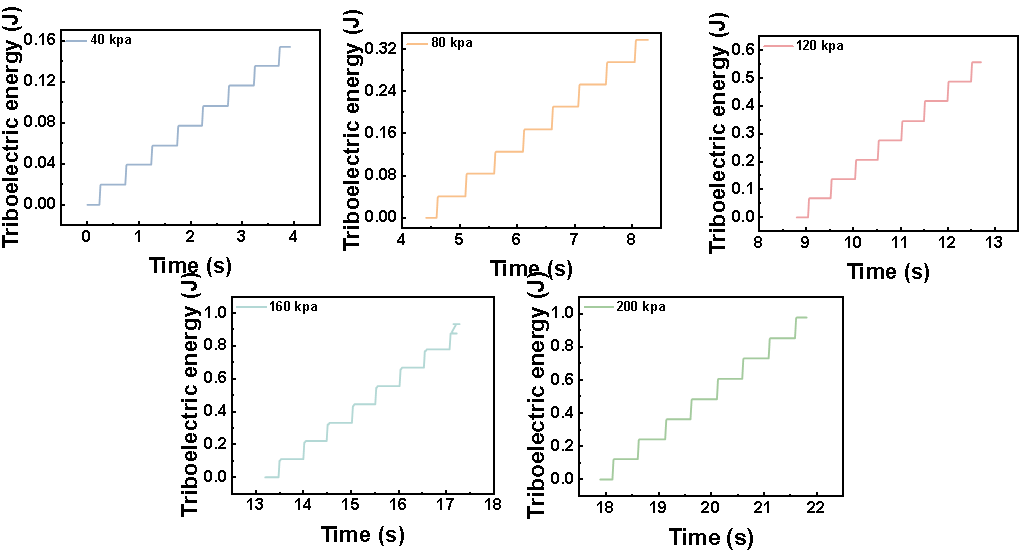


**Fig. S3** **Temporal evolution of the triboelectric output from the Auxetic-TENG under applied pressures of 40 kPa, 80 kPa, 120 kPa, 160 kPa, and 200 kPa**


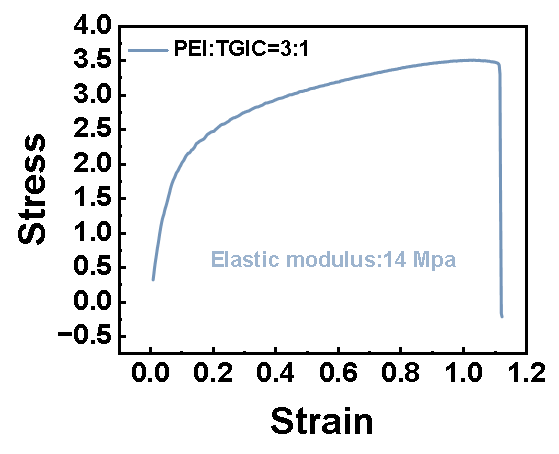


**Fig. S4** **Stress-strain curve and elastic modulus of the PEI/TGIC blend at the optimized ratio of 3:1**


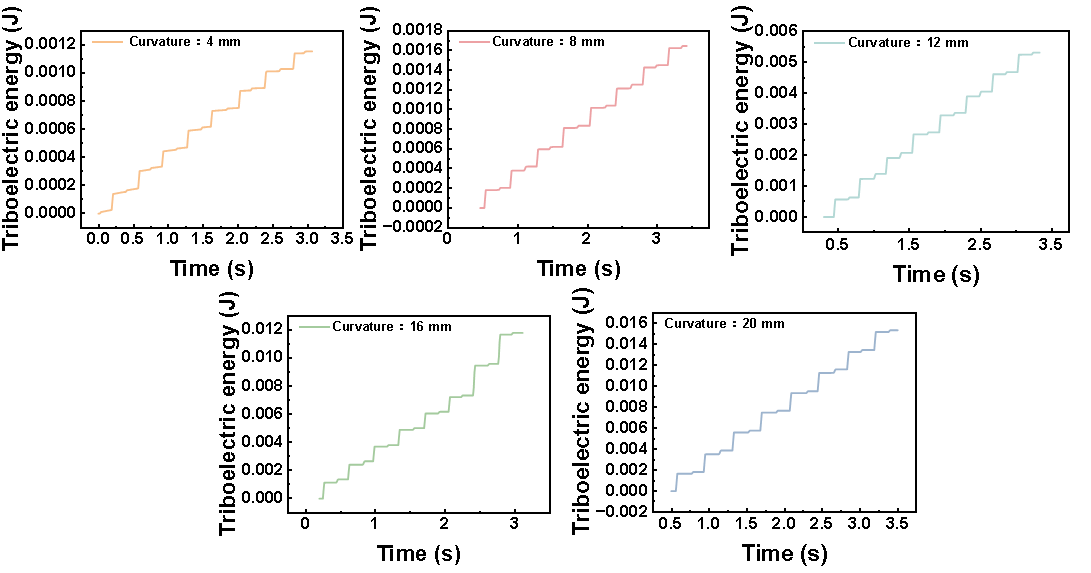


**Fig. S5** **Temporal evolution of the triboelectric output from the Auxetic-TENG under bending displacements ranging from 4 mm to 20 mm**


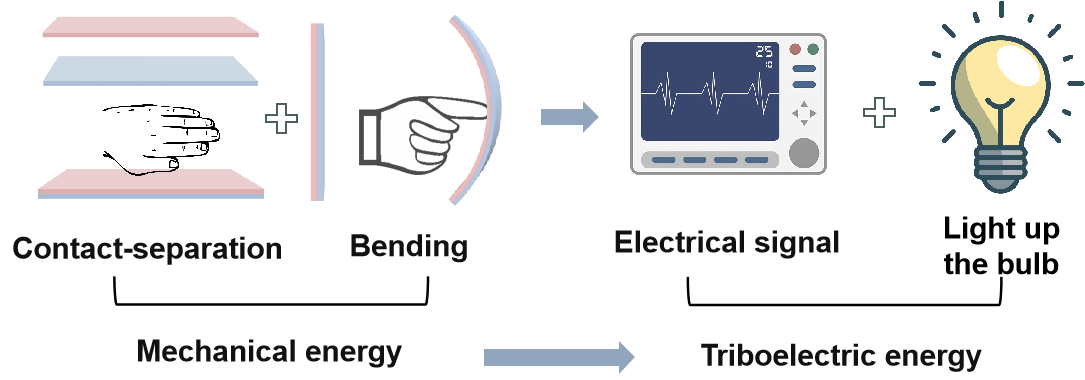


**Fig. S6 Conversion of applied mechanical energy into triboelectric output in the Auxetic-TENG during contact-separation and bending modes for signal discrimination and light emission applications**


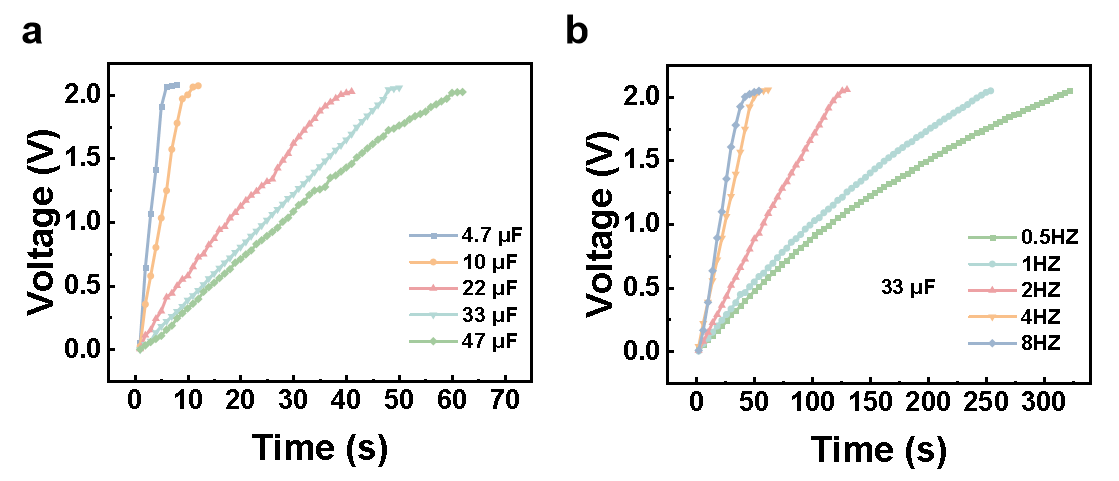


**Fig. S7** **Charging characteristics of the Auxetic-TENG: (a) Charging capacitors of different capacitances (4.7 µF, 10 µF, 22 µF, 33 µF, 47 µF) to saturation; (b) Charging a 33 µF capacitor at different operating frequencies (0.5 Hz, 1 Hz, 2 Hz, 4 Hz, 8 Hz)**


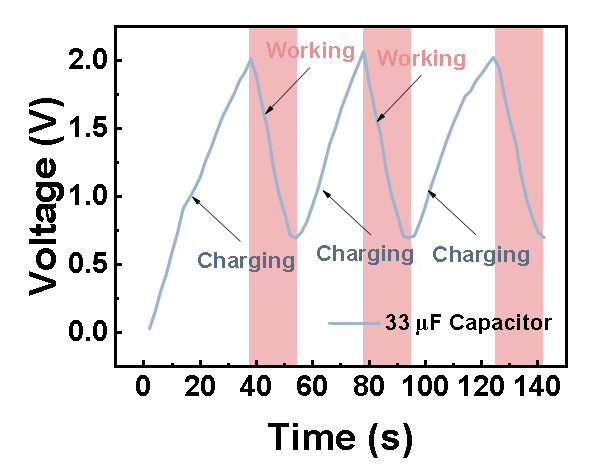


**Fig. S8 Charging and discharging of a timer by the Auxetic-TENG**


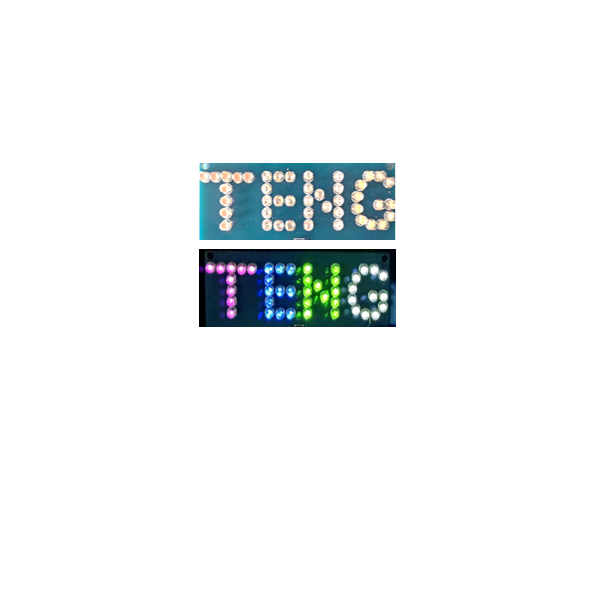


**Fig. S9 Powering of 44 light-emitting diodes (LEDs) by the Auxetic-TENG**


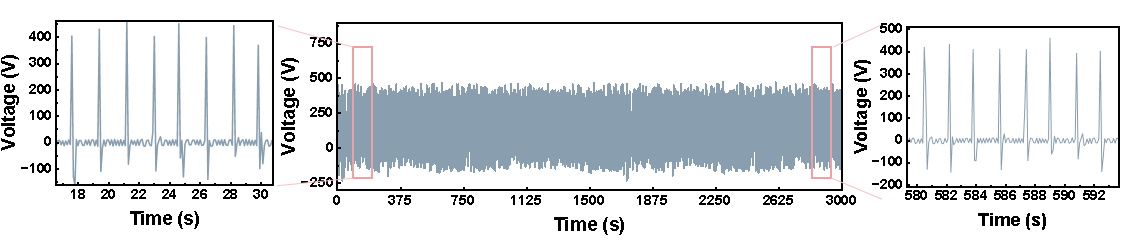


**Fig. S10** **Stability of the Auxetic-TENG's output signal during continuous operation for 1 h**

**
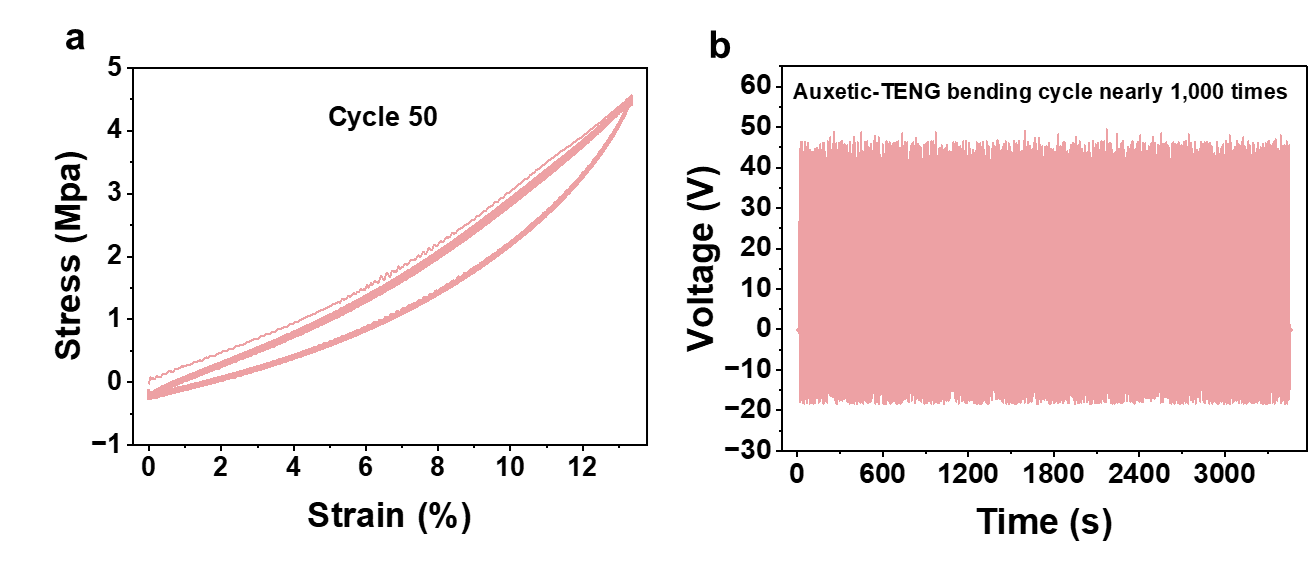
**

**Fig. S11** Operational Stability of Auxetic-TENG. (**a**) Mechanical durability of the negative Poisson's ratio structure after 50 tensile cycles. (**b**) Operational stability after 1000 bending cycles of Auxetic-TENG


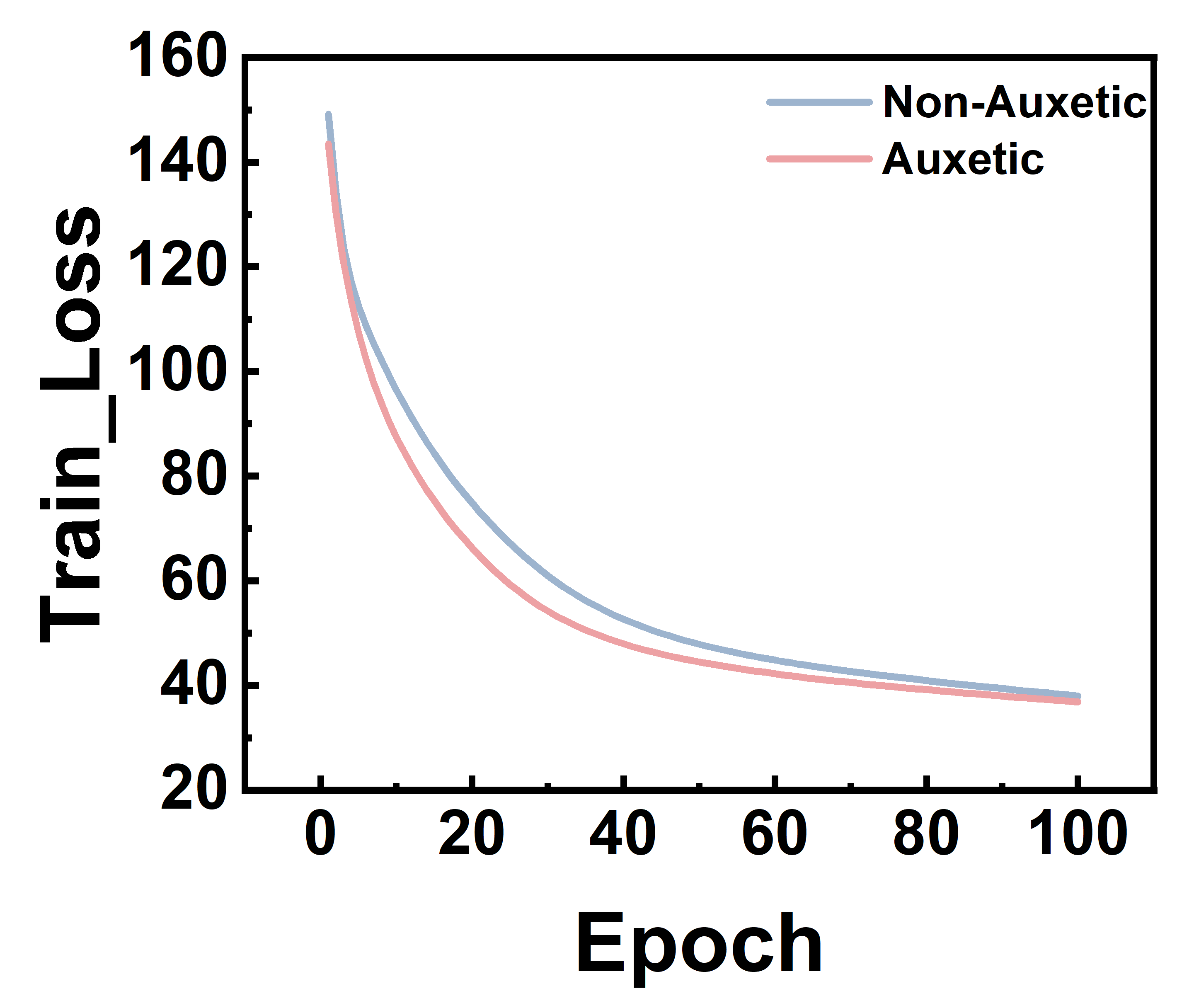


**Fig. S12** The learning loss of FCNN varying over time


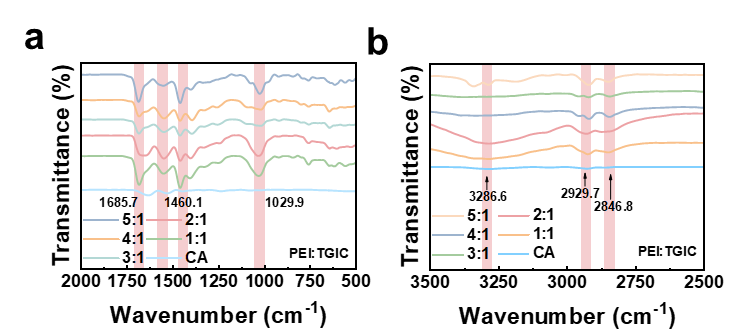


**Fig. S13** Infrared spectra of the positive tribo-material (CA/PEI) of the Auxetic-TENG at different modification ratios


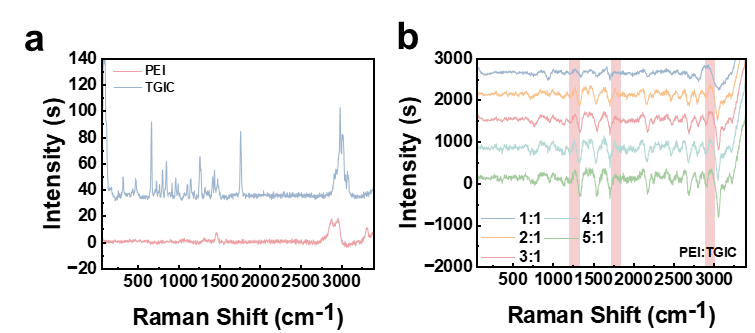


**Fig. S14 a** Raman point mapping analysis of PEI and TGIC. **b** Raman point mapping of CA/PEI at different blend ratios


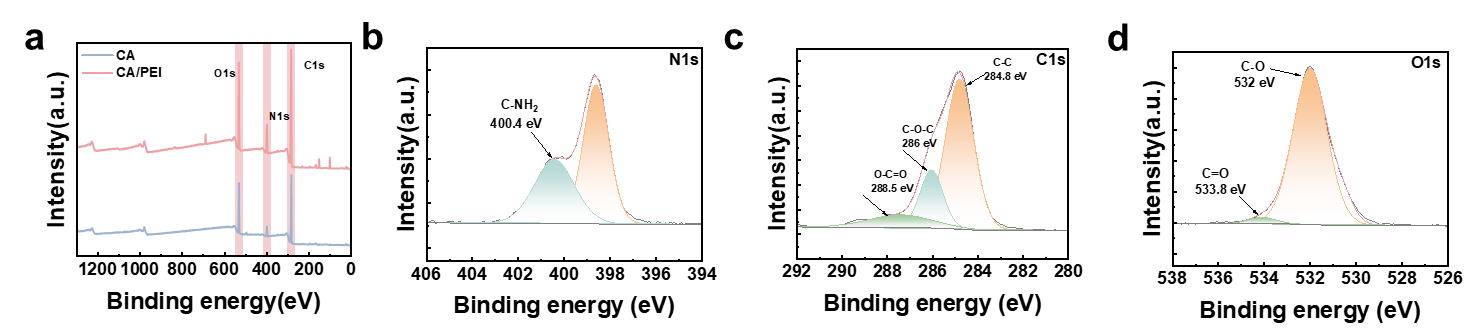

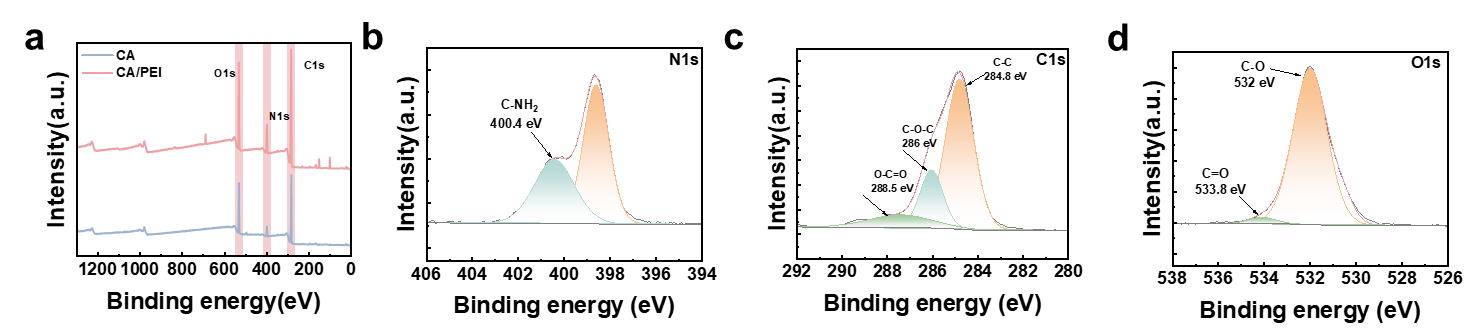


**Fig. S15 a** XPS survey spectra of CA and CA/PEI. **b-d** High-resolution XPS deconvolution of CA/PEI: (b) N 1s, (c) O 1s, (d) C 1s core levels


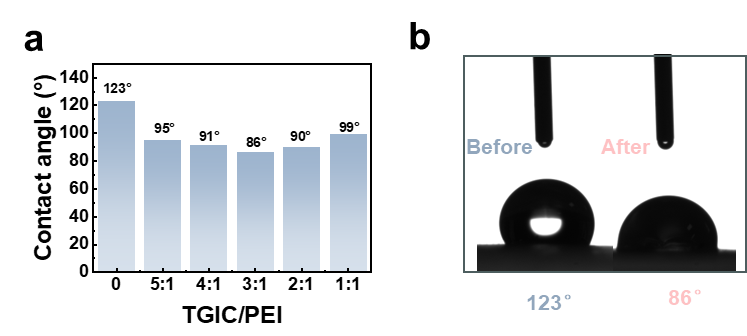


**Fig. S16 a** Water contact angle measurements of CA and CA/PEI at varying compositions. **b** Representative optical images of water contact angles on pristine CA and modified CA/PEI surfaces


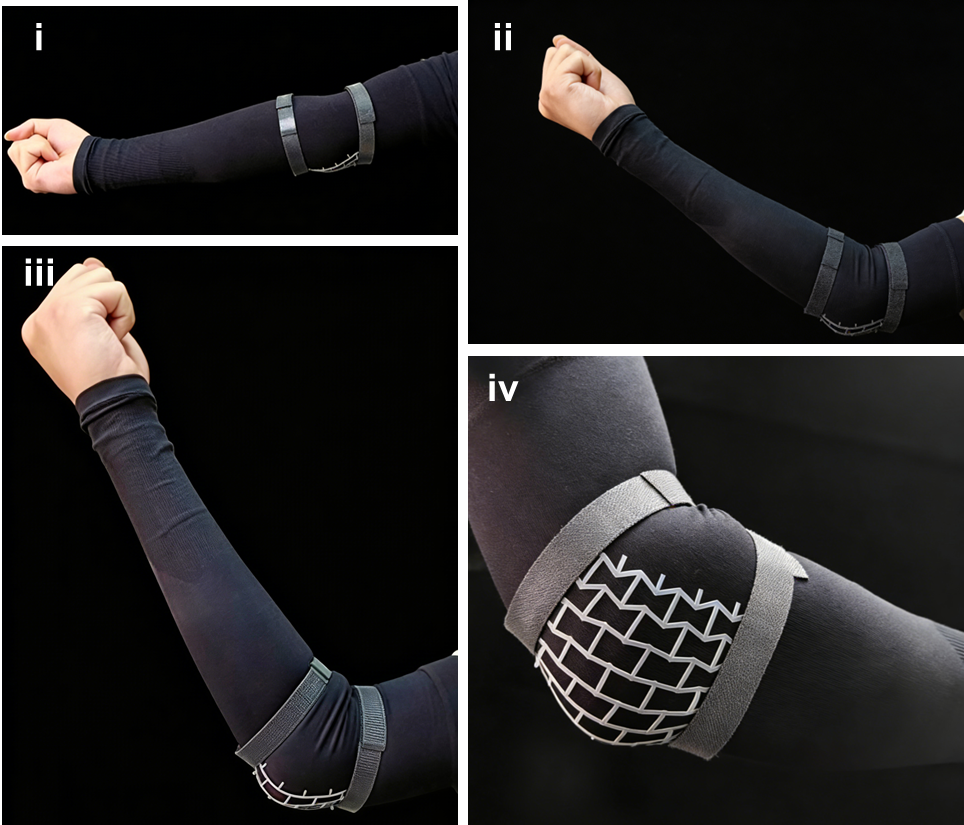


**Fig. S17** Demonstration of the synclastic curvature effect of the auxetic material on a human elbow joint. (i) Photograph of the material on an extended arm. (ii) Photograph during moderate flexion of the elbow. (iii) Photograph during significant flexion, where the auxetic structure naturally forms a dome-like shape to match the joint's convexity, preventing edge lifting. (iv) Detailed view of the conformed auxetic network under bending load.


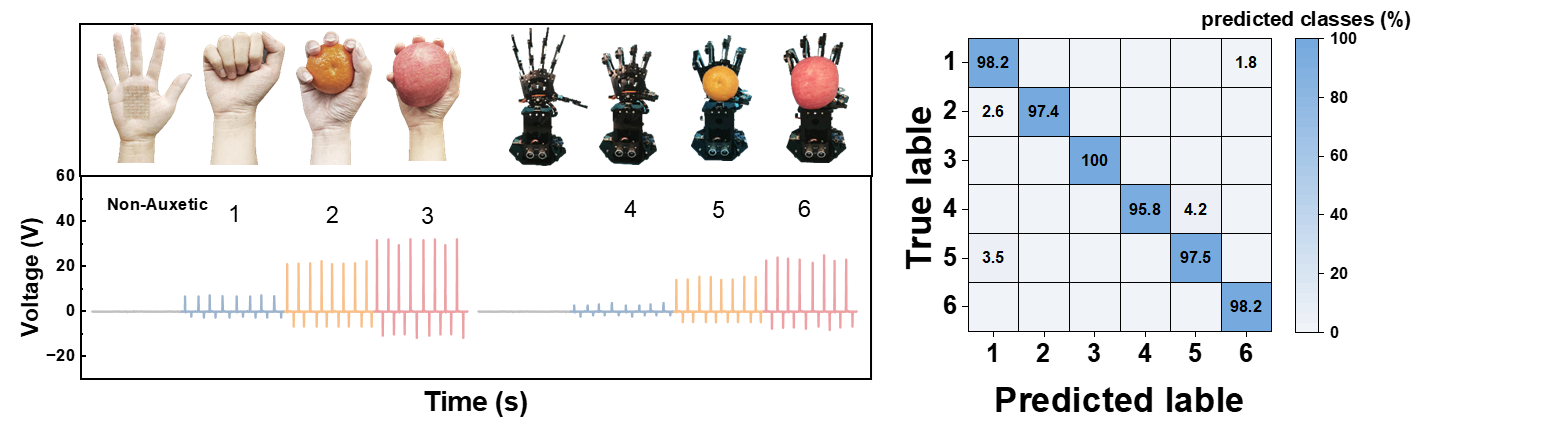


**Fig. S18** Non-Auxetic-TENG achieves 97% recognition accuracy after CNN deep learning for input signals from human hands and robotic grippers grasping different objects


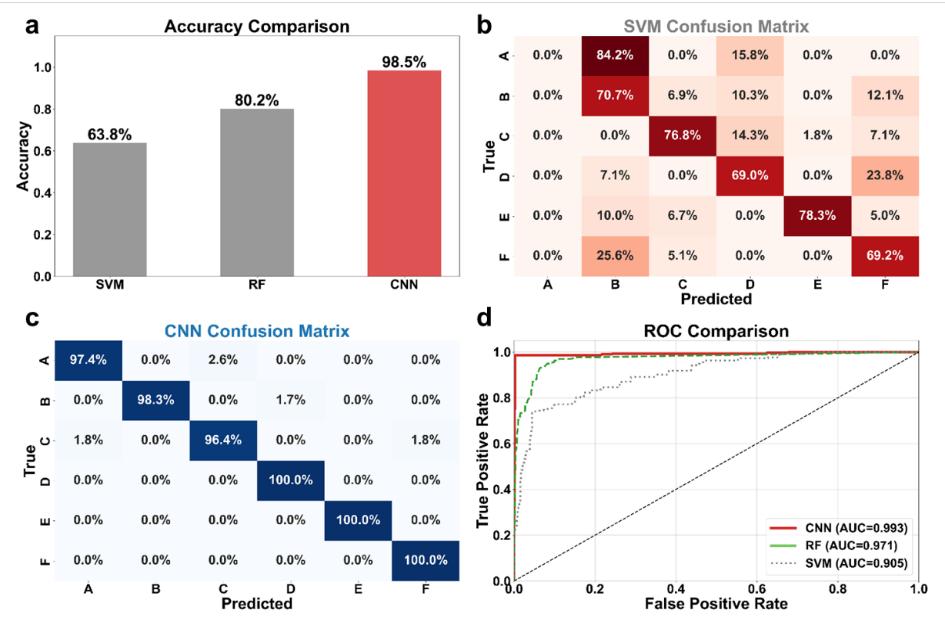


**Fig. S19** Quantitative performance comparison between conventional machine learning algorithms and the proposed CNN framework under identical experimental conditions. (**a**) Overall classification accuracy comparison, revealing that the CNN model (98.5%) significantly outperforms Support Vector Machine (SVM, 63.8%) and Random Forest (RF, 80.2%). (**b**) Normalized confusion matrix for the SVM model, exhibiting considerable off-diagonal misclassification (red blocks) due to the inability to resolve feature ambiguity among similar signal patterns. (**c**) Normalized confusion matrix for the CNN model, demonstrating a clear diagonal dominance with minimal error, confirming its capability to extract distinct spatiotemporal fingerprints. (**d**) Receiver Operating Characteristic (ROC) curve comparison. The CNN model achieves the highest Area Under Curve (AUC = 0.993), enclosing the RF (0.971) and SVM (0.905) curves, thereby validating its superior sensitivity and robustness against signal variability.

**Supplementary Tables**

**Table S1** The energy conversion efficiency of Auxetice-TENG during contact and separation under different pressures [S4, S5]

| Pressure | Mechanical Energy  (E_in_=E_1_+E_2_) | Triboelectric Energy  E_out_ | Conversion Efficiency  η_1_ |
| --- | --- | --- | --- |
| 40 kpa | 0.2 J+1.47×10^-4^ J | 0.0197 J | 9.84% |
| 80 kpa | 0.4 J+1.47×10^-4^ J | 0.0405 J | 10.12% |
| 120 kpa | 0.6 J+1.47×10^-4^ J | 0.0687 J | 11.45% |
| 160 kpa | 0.8 J+1.47×10^-4^ J | 0.1105 J | 13.8% |
| 200 kpa | 1 J+1.47×10^-4^ J | 0.1226 J | 12.25% |

**Table S2** The energy conversion efficiency of Auxetice-TENG at different bending displacements

| Bending  displacement | Mechanical Energy  E_in_ | Triboelectric Energy  E_out_ | Conversion Efficiency  η_2_ |
| --- | --- | --- | --- |
| 4 mm | 0.000564 J | 0.0001773 J | 31.3% |
| 8 mm | 0.000826 J | 0.000204 J | 24.7% |
| 12 mm | 0.0039 J | 0.000632 J | 16.2% |
| 16 mm | 0.011 J | 0.00137 J | 12.45% |
| 20 mm | 0.024 J | 0.00182 J | 7.58% |

**Table S3** The energy conversion efficiency of Non-Auxetice-TENG at different bending displacements

| Bending  displacement | Mechanical Energy  E_in_ | Triboelectric Energy  E_out_ | Conversion Efficiency  η_3_ |
| --- | --- | --- | --- |
| 4 mm | 0.000103 J | 0.0000301 J | 29.15% |
| 8 mm | 0.00155 J | 0.0001128 J | 7.28% |
| 12 mm | 0.00734 J | 0.000352 J | 4.8% |
| 16 mm | 0.0195 J | 0.0007469 J | 3.83% |
| 20 mm | 0.04423 J | 0.001048 J | 2.37% |

**Table S4** Detailed configuration and hyperparameters of the proposed CNN architecture

| Layer Type | Output Shape (Channels/Units) | Kernel Size / Stride | Activation Function |
| --- | --- | --- | --- |
| Input | 1×L (Time-series) | - | - |
| Convolution 1 (C1) | 6 | 5/1 | ReLU |
| Convolution 2 (C2) | 12 | 5/1 | ReLU |
| Convolution 3 (C3) | 60 | 5/1 | ReLU |
| Flatten | - | - | - |
| Fully Connected 1 (FC1) | 60 | - | ReLU |
| Fully Connected 2 (Output) | 6 | - | Softmax |

**Table S5** Training hyperparameters used in this study

| Hyperparameter | Value |
| --- | --- |
| Optimizer | Adam (Adaptive Moment Estimation) |
| Learning Rate | 0.001 |
| Loss Function | Cross-Entropy Loss |
| Batch Size | 32 |
| Total Epochs | 100 |
| Framework | PyTorch |

**Supplementary References**

1. M.S. Kim, Y. Lee, J. Ahn, S. Kim, K. Kang et al., Skin-like omnidirectional stretchable platform with negative Poisson’s ratio for wearable strain–pressure simultaneous sensor. Adv. Funct. Mater. **33**(3), 2208792 (2023). <https://doi.org/10.1002/adfm.202208792>
2. S. Dong, H. Hu, Sensors based on auxetic materials and structures: a review. Materials **16**(9), 3603 (2023). <https://doi.org/10.3390/ma16093603>
3. H. Wu, C. Shan, S. Fu, K. Li, J. Wang et al., Efficient energy conversion mechanism and energy storage strategy for triboelectric nanogenerators. Nat. Commun. **15**(1), 6558 (2024). <https://doi.org/10.1038/s41467-024-50978-7>
4. Y.-C. Wang, M.-W. Shen, S.-M. Liao, Microstructural effects on the Poisson’s ratio of star-shaped two-dimensional systems. Phys. Status Solidi B **254**(12), 1770264 (2017). <https://doi.org/10.1002/pssb.201770264>
5. R. Zhang, M. Hummelgård, J. Örtegren, M. Olsen, H. Andersson et al., Interaction of the human body with triboelectric nanogenerators. Nano Energy **57**, 279–292 (2019). <https://doi.org/10.1016/j.nanoen.2018.12.059>
